# Noise reduction as an emergent property of single-cell aging

**DOI:** 10.1038/s41467-017-00752-9

**Published:** 2017-09-25

**Authors:** Ping Liu, Ruijie Song, Gregory L. Elison, Weilin Peng, Murat Acar

**Affiliations:** 1 Department of Molecular Cellular and Developmental Biology, Yale University, 219 Prospect Street, New Haven, CT 06511 USA; 2 Systems Biology Institute, Yale University, 850 West Campus Drive, West Haven, CT 06516 USA; 3 Interdepartmental Program in Computational Biology and Bioinformatics, Yale University, 300 George Street, Suite 501, New Haven, CT 06511 USA; 4 Department of Physics, Yale University, 217 Prospect Street, New Haven, CT 06511 USA

## Abstract

Noise-induced heterogeneity in gene expression is an inherent reality for cells. However, it is not well understood how noise strength changes for a single gene while the host cell is aging. Using a state-of-the-art microfluidic platform, we measure noise dynamics in aging yeast cells by tracking the generation-specific activity of the canonical *GAL1* promoter. We observe noise reduction during normal aging of a cell, followed by a short catastrophe phase in which noise increased. We hypothesize that aging-associated increases in chromatin state transitions are behind the observed noise reduction and a stochastic model provides quantitative support to the proposed mechanism. Noise trends measured from strains with altered *GAL1* promoter dynamics (constitutively active, synthetic with nucleosome-disfavoring sequences, and in the absence of *RPD3*, a global remodeling regulator) lend further support to our hypothesis. Observing similar noise dynamics from a different promoter (*HHF2*) provides support to the generality of our findings.

## Abstract (web summary)

Gene expression is a noisy process, but it is not known how noise in gene expression changes during the aging of single cells. Here the authors show that noise decreases during normal aging, and provide support for aging-associated increases in chromatin state transitions governing noise reduction.

## Introduction

Owing to the inherently stochastic nature of molecular interactions, isogenic cells grown in the same environment display differences in their gene expression levels. Such differences, known as variability or noise in gene expression, are introduced at various regulatory steps including chromatin remodeling, transcription, translation, and posttranslational modifications^1–4^. Depending on the cellular system it is associated with, noise can be beneficial or detrimental to the progression of cellular activities^5, 6^. For example, activities requiring precision in the number of expressed proteins would suffer from noise^5^, whereas noise-induced phenotypic switching can be beneficial to population fitness by facilitating a bet-hedging strategy in fluctuating environments^6^.

Noisy gene expression in single cells has been experimentally characterized in several different contexts including development^7, 8^, metabolism^3, 9, 10^, and stress response^11, 12^. However, aging has been an exception due in large part to the technical challenges associated with quantifying noise from aging cells until the end of their lifespan. For instance, the conventional micromanipulator-based aging assays lacked the capacity to measure gene expression levels simultaneously with life span^13, 14^. How do the fluctuations in the expression of a single gene change while a cell is aging? What dictates the degree of such stochastic fluctuations? Despite the fundamental nature of these questions, we have little to no empirical understanding into how cellular aging affects noise in gene expression in a single cell.

Here we measure noise dynamics in aging yeast cells with a state-of-the-art microfluidic platform by tracking the generation-specific activity of the canonical *GAL1* promoter (P_*GAL1*_) throughout the replicative lifespan of the cells and observe noise reduction during normal aging of a cell, followed by a short catastrophe phase in which noise increased. Assisted by a stochastic model, we hypothesize that aging-associated increases in chromatin state transitions are behind the observed noise reduction. The noise trends we measure from various yeast strains with significantly different P_*GAL1*_ dynamics lend further support to our hypothesis. Moreover, supporting the generality of our findings, we are able to observe similar noise dynamics from a different promoter (*HHF2*).

## Results

### Measuring single-cell gene expression levels during aging

**Fig. 1:**
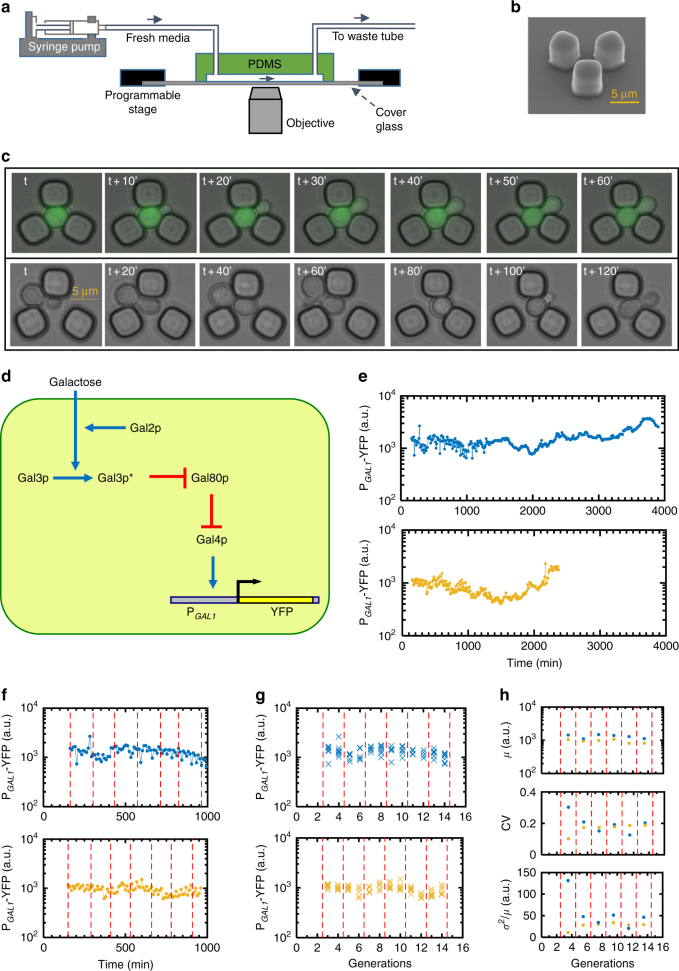
Experimental setup, galactose network, and single-cell fluorescence trajectories. **a** Schematics of the experimental setup. **b** SEM picture of a single replicator unit. *Scale bar*, 5 µm. **c** Sample time-lapse microscopy snapshots covering one cell generation (*top*) and illustrating the initial trapping of virgin cells (*bottom*). *Scale bar*, 5 µm. **d** Network architecture built by the four regulatory genes. The galactose-bound state of Gal3p is denoted by Gal3p*. *Pointed blue arrows* reflect activation and *blunt red arrows* reflect inhibition. **e** Two sample single-cell fluorescence trajectories in chronological order. Using cells of the wild-type strain, fluorescence level is measured every 10 min. **f**–**h** Illustration of analysis procedure. The *red dashed lines* indicate the boundaries of two-generation windows. **f** Chronological fluorescence measurements for the initial 1,000 min of the cells shown in **e**. **g** Chronological fluorescence measurements in **f** are assigned to the corresponding generations. Each *cross mark* represents one fluorescence measurement in that generation. **h** For each cell in **g**, the measurements within each two-generation window are used to calculate the mean, CV, and Fano factor of expression levels within that window for that cell

To gain quantitative insights into the dynamics of gene expression noise during cellular aging, we measured gene expression levels in replicatively aging yeast cells using a microfluidic platform^15^ facilitating the real-time counting of generation numbers for the tracked mother cells (Fig. 1a–c), which are trapped as virgin cells as they separate from their own mother (Fig. 1c). Replicative life span (RLS)^13, 14, 16^ is a commonly used aging metric quantifying the number of daughter cells a mother produces until it dies. As the genetic system, we chose to measure the activity and noise from the canonical galactose network (GAL network)^3, 6, 9, 10^, whose activity is reported by a P_*GAL1*_-YFP construct we integrated into the haploid yeast genome (Fig. 1d). The P_*GAL1*_ reflects the activity of the entire GAL network due to the presence of Gal4-binding sites on the promoter. The cascade of molecular interactions starting from galactose uptake by Gal2 and other transporters transmit the galactose signal to the Gal4 transcription factor^9, 10, 17, 18^. The activation of the inducer Gal3 by galactose and the binding of active Gal3 proteins to the repressor Gal80 compose the intermediate steps of this signaling cascade. When Gal80 repressors are bound by active Gal3 inducers, they can no longer repress Gal4 activators, turning on transcription from the P_*GAL1*_ carrying the active Gal4 proteins.

Bright-field and fluorescence images of the trapped mother cells were captured time dynamically. The bright-field images were taken every 10 min to facilitate the quantification of generation times. Yellow fluorescent protein (YFP) snapshots were also taken every 10 min, an interval chosen to minimize phototoxicity effects. As a result, each mother cell was probed using four to nine YFP snapshots per generation; longer generation times contained more YFP snapshots. Taking multiple fluorescence measurements per generation throughout different cell cycle stages allowed us to minimize errors, including those introduced by potential cell-cycle effects.

The fluorescence values measured during each generation were averaged and the average value was used as the representative network activity level for each generation of a specific mother cell. Figure 1e, f illustrates how the activity of the wild type GAL network changes in a single cell during the aging process. The cell displayed time-dynamic variations in network activity due to the stochastic nature of the gene expression steps.

The wild-type cells displayed an average lifespan of 22.9 generations (Supplementary Fig. 1). Naturally, there was variation among the cells in terms of their replicative lifespan. Some cells lived only 4 generations, whereas others were alive until 53 generations.

### Generation-specific noise dynamics of P_*GAL1*_ during aging

**Fig. 2:**
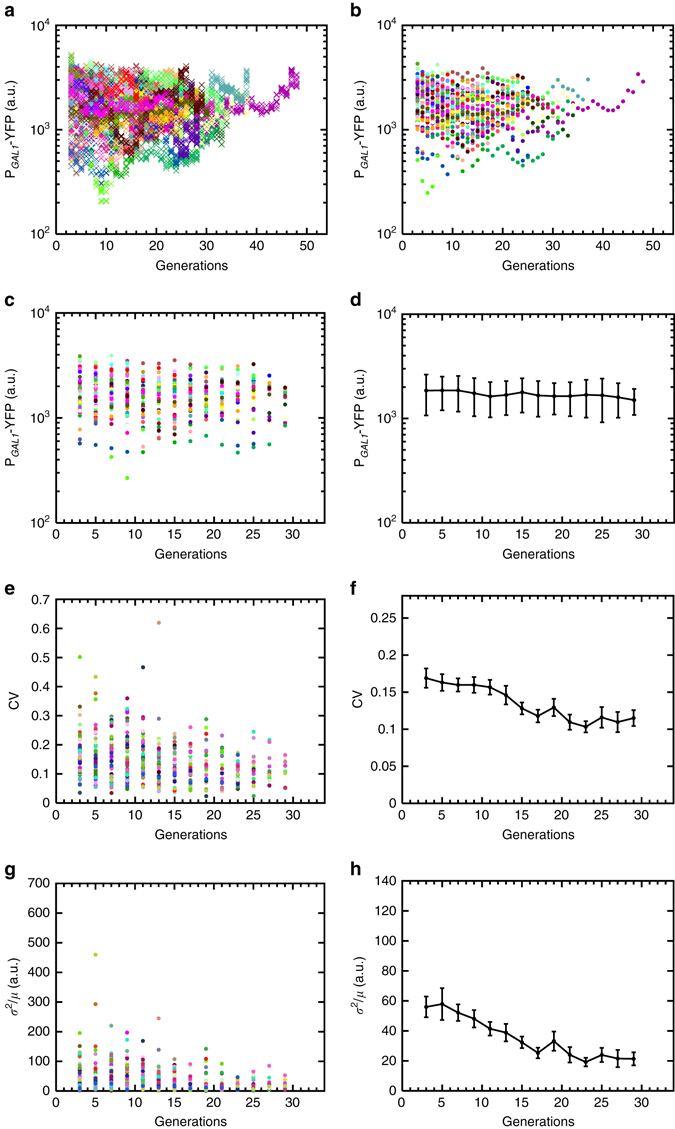
Generation-specific activity of the *GAL1* promoter in wild-type background (strain yTY10a) and the resulting noise dynamics during aging. **a** Generational fluorescence levels for *N* = 59 wild-type ON mother cells analyzed in this study. Each generation has four measurements or more. All the mother cells used in the study have replicative lifespan of 10 generations or more. **b** Mean generational fluorescence levels for the cells described in **a**. **c** The aging axis is divided into non-overlapping windows of two generations. The mean fluorescence levels inside these windows are plotted for each individual cell. **d** Mean and SD of fluorescence levels across all available cells inside each window. The *error bars* denote SD, the number of data points used for the SD quantification are 10 or above. **e** CV values of individual cells inside each window. **f** Mean and SEM of the CV’s across the cell population as shown in **e**. **g** Fano factor values of individual cells inside each window. **h** Mean and SEM of the Fano factors across the cell population as shown in **g**. For the SEM quantifications in **f**, **h**, the number of data points used is 10 and above

We measured the variability in gene expression using two noise metrics^1, 4^: the coefficient of variation (CV), defined as the SD divided by the mean (*σ*/*μ*); and the Fano factor, defined as the variance divided by the mean (*σ* ^2^/*μ*). As a reliable measurement of variance or standard deviation requires a sufficiently large sample size, separately for each mother cell, we partitioned our single-cell YFP readings (Figs. 1e and 2a, b) into non-overlapping windows of two consecutive generations (Figs. 1f, g and 2c, d) and quantified the noise value for each mother cell over each two-generations window before averaging these single-cell noise values across all mother cells falling into each such window (Figs. 1h and 2e–h). Noise in the activity of the wild type GAL network decreased significantly during aging. The CV decreased by ~35% while the Fano factor displayed a ~57% decrease (Fig. 2e–h). Most of this noise reduction occurred in the first 16 generations, after which the noise profiles reached a plateau.

### Noise dynamics of constitutively active P_*GAL1*_ in aging cells

**Fig. 3:**
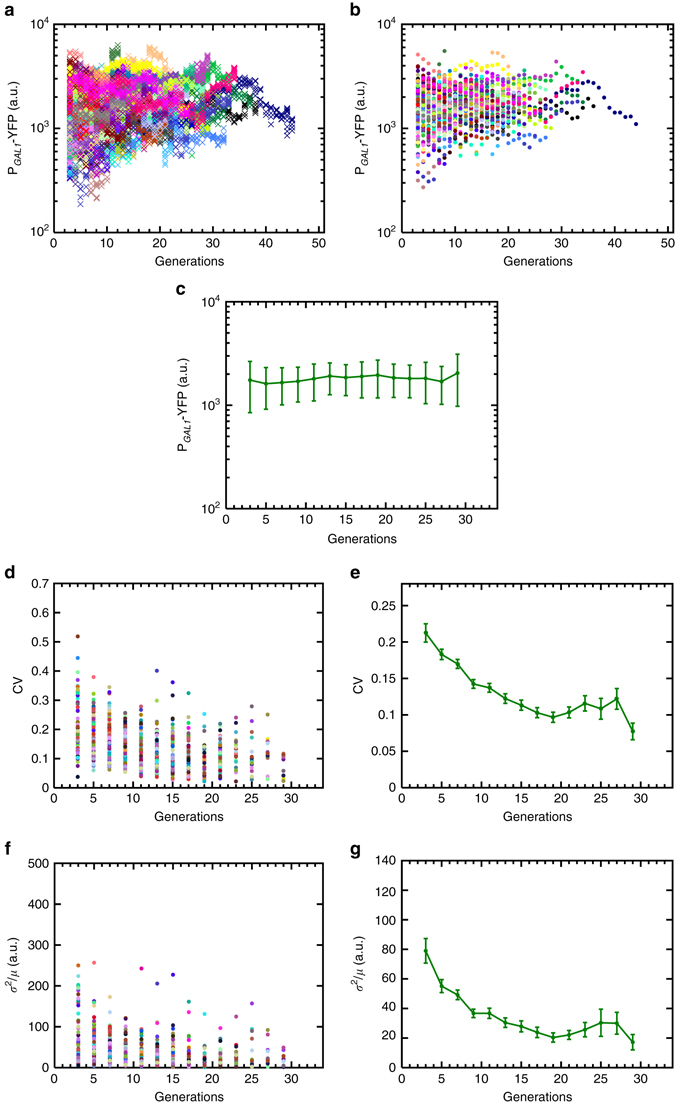
Generation-specific activity of the *GAL1* promoter in *gal80*Δ background (strain WP190) and the resulting noise dynamics during aging. **a** Generational fluorescence levels for *N* = 102 ON mother cells of the *GAL80* gene deleted strain. Each generation has four measurements or more. All the mother cells used in the study have replicative lifespan of 10 generations or more. **b** Mean generational fluorescence levels for the cells described in **a**. **c** The aging axis is divided into non-overlapping windows of two generations, and the mean and SD of fluorescence levels across all available cells inside each window are plotted. The *error bars* denote SD, the number of data points used for the SD quantification are 10 or above. **d** CV values of individual cells inside each window. **e** Mean and SEM of the CV’s across the cell population as shown in **d**. **f** Fano factor values of individual cells inside each window. **g** Mean and SEM of the Fano factors across the cell population as shown in **f**. For the SEM quantifications in **e**, **g**, the number of data points used is at least 10

How can we dissect the aging-associated noise reduction observed from the wild type GAL network activity in terms of contributions from the aging effects on the P_*GAL1*_ and on the upstream regulatory components of the network affected by the aging process? The P_*GAL1*_-reported activity of the GAL network was shown^10, 19, 20^ to be compensated against network-level coordinated changes in the expression of the upstream regulatory components. Therefore, if aging affects gene expression levels from all network promoters in similar proportions, it would effectively constitute a form of network-level coordinated changes, and so the net regulatory effects of the upstream components would be unchanged due to compensation, and the observed noise reduction from the P_*GAL1*_ would be solely due to aging-associated changes on the P_*GAL1*_ itself. To discriminate between these two models, we cut the connection between the P_*GAL1*_ and the upstream regulatory cascade by deleting the *GAL80* gene from the yeast genome, resulting in a constitutively ON expression profile from the promoter (Supplementary Fig. 2). Single cells still displayed noise reduction while they were aging (Fig. 3), indicating that, in the wild type network, the effect of the upstream regulatory components on the downstream reporter’s noise profile is compensated. The extent of noise reduction in the *GAL80* deletion strain was higher than that in wild type (compare Fig. 3e, g with Fig. 2f, h), principally due to a higher initial noise level in the *GAL80* deletion strain. We attributed this observation to the loss of the noise-reducing effects of the *GAL80-*mediated negative feedback loop.

### Mechanistic insights from a stochastic model

**Fig. 4:**
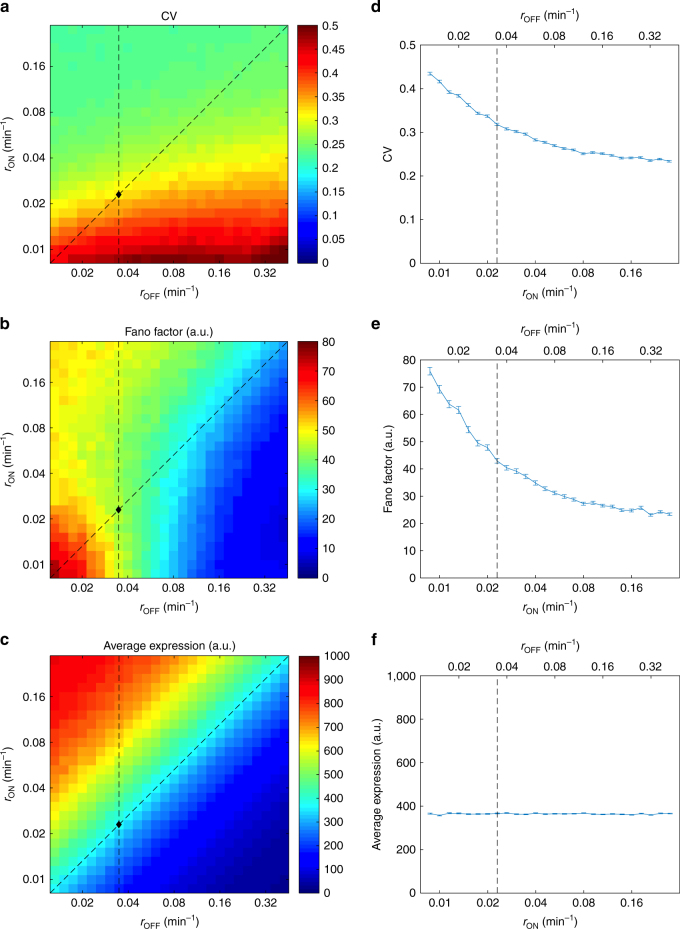
Quantitative model predictions for the noise and gene expression dynamics during aging from the *GAL1* promoter in *gal80*Δ background. **a**–**c** Effect of *r*_ON_ and *r*_OFF_ on noise in expression (measured by CV **a** and Fano factor **b**) and on average expression level **c**. The *black diamond* indicates the fitted parameter values of the native *GAL1* promoter (*r*_OFF_  = 0.0348 min^−1^, *r*_ON_  = 0.0229 min^−1^). The *vertical dashed line* indicates the range of parameter values used for the simulations in Supplementary Fig. 3, where *r*_OFF_ is kept constant. The *diagonal dashed line* inicates the range of parameter values used for the simulations in **d**–**f**, where the average expression level is kept constant by varying the value of *r*_OFF_ correspondingly. **d**–**f** Effect of *r*_ON_ and *r*_OFF_ on noise in expression (measured by CV **d** and Fano factor **e**) and on average expression level **f** when the average expression level is kept approximately constant by varying the two parameters at the same ratio. The *dashed line* indicates the fitted parameter values of the native *GAL1* promoter (*r*_OFF_  = 0.0348 min^−1^, *r*_ON_ 0.0229 min^−1^). *Error bars* indicate SEM (*N* = 2000)

These results obtained from the constitutive P_*GAL1*_ elucidated how noise from a single gene is shaped by aging-associated changes occurring in cells. What causes a cell to reduce the amount of variation in gene expression during aging? Chromatin remodeling, involving transitions between the open and closed forms of the chromatin, is known to be a major contributor to gene expression noise in eukaryotes^3, 4, 21^. To quantitatively evaluate how changes in the transition rates between the OFF and ON states of the P_*GAL1*_ could explain the observed noise decrease during aging, we used a detailed stochastic model^10, 22^ incorporating various stages of eukaryotic gene expression time dynamically (Supplementary Note 1). Using our model, we first extracted the values of promoter transition rates (*r*_ON_ and *r*_OFF_) for the constitutive P_*GAL1*_ in young cells. Based on our assumption that the aging process changes noise by altering the *r*_ON_ and *r*_OFF_ rates in single cells, we systematically varied these rates to mimick how aging impacts them and calculated the resulting noise values (Fig. 4a–c).

We saw that only certain trajectories of changes in *r*_ON_ and *r*_OFF_ were allowed if noise were to decrease and YFP expression levels were to display specific trends. For example, cells could lower noise in *GAL1* activity during aging if they kept *r*_OFF_ to be the same, while *r*_ON_ is increasing. Yet another path of the aging process that could lead to noise reduction was if it increased the *r*_ON_ and *r*_OFF_ values in equal proportions. Despite impacting the noise dynamics similarly, these two paths would lead to different expression level changes during aging. With the first path, increasing *r*_ON_ values would make the promoter spend more time in the ON state, increasing transcription and hence the number of YFP molecules produced as cells age. The extreme stability of YFP (and hence extremely minimal active degradation) means that YFP concentrations are reduced principally by dilution and the total amount of volume increase per cell cycle is approximately constant: the size of the mother compartment is known to increase linearly as cells age^23^ and we observed similar daughter cell sizes during the early portion of the replicative lifespan, where the noise reduction is most prominent. Thus, the number of new YFP molecules per generation required to maintain the same YFP concentration as cells grow should also be roughly constant and the cell size increase alone cannot offset the increased expression from the promoter. We therefore would expect the observed YFP concentration to increase as well in the first path (Supplementary Fig. 3). The second path, on the other hand, would reduce noise while keeping the expression level at similar values throughout aging (Fig. 4c, f). To distinguish between these two cases, we measured single cell YFP expression levels from the constitutive P_*GAL1*_ during aging. We found the expression levels to display a flat profile while cells were aging (Fig. 3c), consistent with the second case discussed above. Therefore, we hypothesize that aging causes similar increases in *r*_ON_ and *r*_OFF_ rates and this leads to the observed noise decrease during aging. Higher *r*_ON_ values could be realized through hardships associated with packing the chromatin into the closed form during aging due to histone depletion in older cells. Indeed, it was shown^24^ that extra histone supply extended yeast lifespan. Alternatively, higher *r*_ON_ values might be due to some cell-size-mediated effect, as older cells are larger in size and it is known that increased cell size result in a higher apparent transcription rate for a large variety of genes^25^, which might be caused by increases in *r*_ON_. The justification for increasing *r*_OFF_ values during aging can be provided through a simpler model that considers aging-associated dysregulation^26, 27^ of the transcriptional machinery, leading to higher transition rates from the ON state to the OFF state.

### Effect of *RPD3* deletion on P_*GAL1*_ noise dynamics

**Fig. 5:**
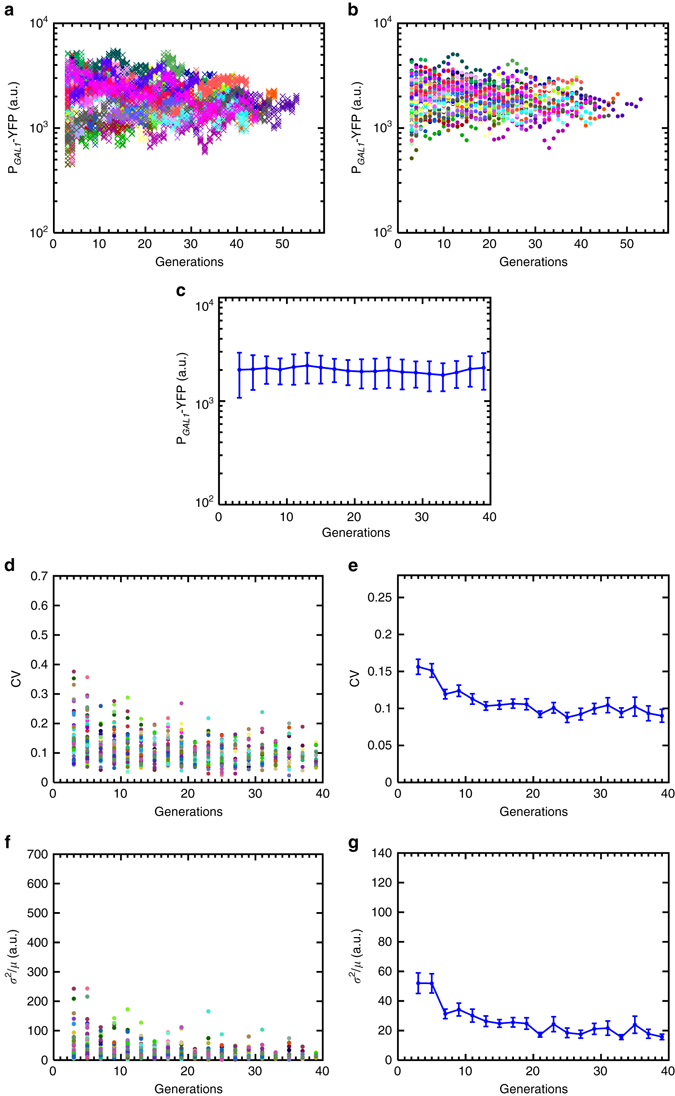
Generation-specific activity of the *GAL1* promoter in *rpd3*Δ background (strain GESC19) and the resulting noise dynamics during aging. **a** Generational fluorescence levels for *N* = 52 ON mother cells of the *RPD3* gene deleted strain. Each generation has four measurements or more. All the mother cells used in the study have replicative lifespan of 10 generations or more. **b** Mean generational fluorescence levels for the cells described in **a**. **c** The aging axis is divided into non-overlapping windows of two generations, and the mean and SD of fluorescence levels across all available cells inside each window are plotted. The *error bars* denote SD, the number of data points used for the SD quantification are 10 or above. **d** CV values of individual cells inside each window. **e** Mean and SEM of the CV’s across the cell population as shown in **d**. **f** Fano factor values of individual cells inside each window. **g** Mean and SEM of the Fano factors across the cell population as shown in **f**. For the SEM quantifications in **e**, **g**, the number of data points used is at least 10

The chromatin remodeling dynamics can be impacted globally in the genome through, e.g., the deletion of remodeling-mediating genes. Rpd3 proteins are well-characterized histone deacetylases whose removal from cells elevates histone levels and extends life span^24^. As another layer of test of our proposed mechanism to explain noise reduction during aging, we deleted the *RPD3* gene from yeast genome and measured the resulting noise dynamics of the wild type P_*GAL1*_ driving YFP (Fig. 5). Single cells lacking the *RPD3* gene lived longer in our microfluidic platform compared with the other strains we characterized (Supplementary Fig. 1). The single-cell YFP expression levels are similar to that of wild-type cells (Figs. 2d and 5c), indicating that *RPD3* likely did not directly affect the chromatin state of the P_*GAL1*_ in our cells. The long-living nature of this strain indicates that it is less susceptible to the detrimental effects of aging, and thus we hypothesized that it would similarly be less prone to whatever aging-related mechanism that is causing increases in *r*_ON_ and *r*_OFF_ as cells age. Indeed, we measured the lowest level of noise change (Fig. 5d–g) from this strain in terms of both CV and Fano factor, compared with the other strains we characterized and presented above. The single-cell YFP expression levels displayed a flat profile during aging (Fig. 5c), suggesting that *r*_ON_ and *r*_OFF_ rates were protected from aging effects in similar proportions.

### Noise dynamics from a synthetically modified P_*GAL1*_

**Fig. 6:**
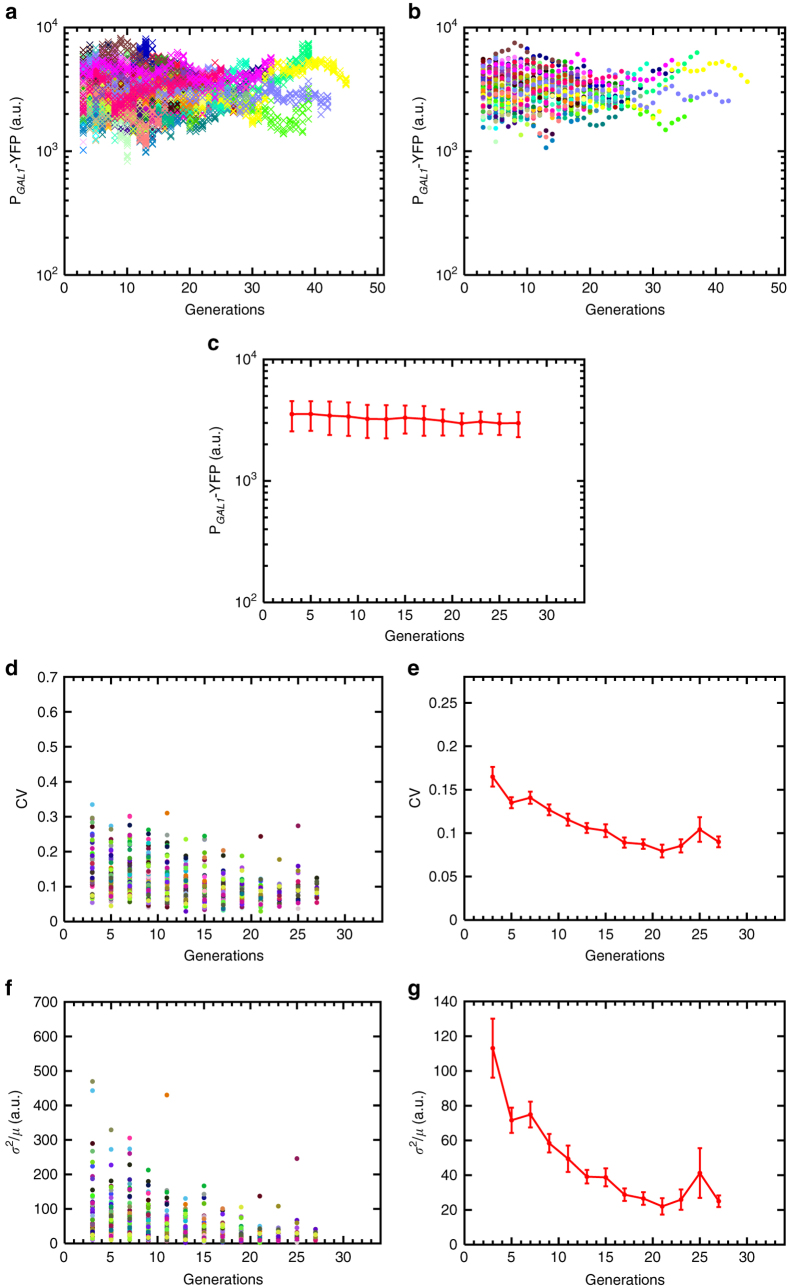
Generation-specific activity of the synthetic *GAL1* promoter in wild-type background (strain GESC11) and the resulting noise dynamics during aging. **a** Generational fluorescence levels for *N* = 73 ON mother cells of the *GAL1* promoter edited strain (GESC11). This strain is edited by introducing nucleosome disfavoring sequences (Methods) onto the *GAL1* promoter driving YFP in the genetic background of the wild-type GAL network. Each generation has four measurements or more. All the mother cells used in the study have replicative lifespan of 10 generations or more. **b** Mean generational fluorescence levels for the cells described in **a**. **c** The aging axis is divided into non-overlapping windows of two generations, and the mean and SD of fluorescence levels across all available cells inside each window are plotted. The *error bars* denote SD, the number of data points used for the SD quantification are 10 or above. **d** CV values of individual cells inside each window. **e** Mean and SEM of the CV’s across the cell population as shown in **d**. **f** Fano factor values of individual cells inside each window. **g** Mean and SEM of the Fano factors across the cell population as shown in **f**. For the SEM quantifications in **e**, **g**, the number of data points used is at least 10

Nucleosome disfavoring sequences^28^ can also alter the dynamics of chromatin state transitions and they can lead to altered dynamics locally on the promoter they are inserted. To further test the remodeling-mediated noise reduction mechanism operating in aging cells, we edited the P_*GAL1*_ by introducing nucleosome disfavoring sequences (Methods) onto it in the genetic background of the wild type GAL network. The genetic edits introduced resulted in higher promoter activity evidenced by higher YFP expression levels (Supplementary Fig. 4). This result was not surprising as the nucleosome disfavoring sequences were expected to increase the average time the promoter was in the open (ON) state. Performing an aging experiment with this strain and analyzing the results, we elucidated a noise reduction profile (Fig. 6d–g), which was similar to the one obtained from the natural P_*GAL1*_ (Fig. 2f), despite the alteration of the chromatin state dynamics in young cells due to promoter editing. This suggests that there is still room for parallel increases in the *r*_ON_ and *r*_OFF_ rates of the synthetic promoter during aging, leading to the decreasing noise pattern and the flat YFP expression profile (Fig. 6c–g).

### No noise reduction in constitutively active synthetic P_*GAL1*_

**Fig. 7:**
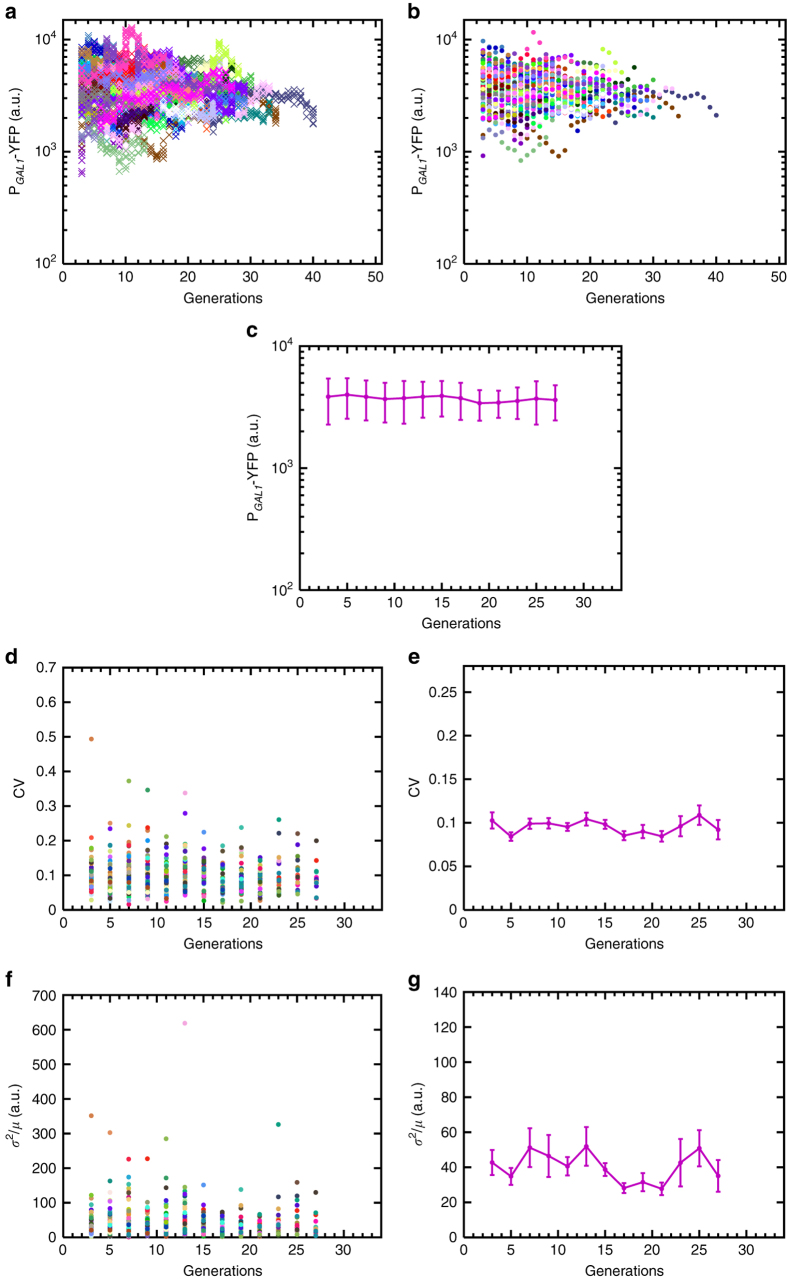
Generation-specific activity of the synthetic *GAL1* promoter in *gal80*Δ background (strain WP274) and the resulting noise dynamics during aging. **a** Generational fluorescence levels for *N* = 82 ON mother cells of the *GAL1* promoter edited and *GAL80* gene deleted strain (WP274). In addition to the deletion of *GAL80* gene, this strain is also edited by introducing nucleosome disfavoring sequences (Methods) onto the *GAL1* promoter driving YFP. Each generation has four measurements or more. All the mother cells used in the study have replicative lifespan of 10 generations or more. **b** Mean generational fluorescence levels for the cells described in **a**. **c** The aging axis is divided into non-overlapping windows of two generations, and the mean and SD of fluorescence levels across all available cells inside each window are plotted. The *error bars* denote SD, the number of data points used for the SD quantification are 10 or above. **d** CV values of individual cells inside each window. **e** Mean and SEM of the CV’s across the cell population as shown in **d**. **f** Fano factor values of individual cells inside each window. **g** Mean and SEM of the Fano factors across the cell population as shown in **f**. For the SEM quantifications in **e**, **g**, the number of data points used is at least 10

Next we hypothesized that, if aging led to higher rates of chromatin remodeling, then a promoter which was already fully open with minimal or no transitions to its OFF state should not display noise reduction during aging as it would already experience a noise ‘floor’ in young cells. To test this hypothesis, we constructed a new strain in which the synthetic P_*GAL1*_ was fused to YFP in a genetic background deleted in the *GAL80* gene. The synthetic P_*GAL1*_ carrying the nucleosome disfavoring sequences had already increased the fraction of time the promoter was in the ON state, however it was necessary to further increase the promoter activity by deleting the transcriptional repressor proteins from the genome. Indeed, the resulting YFP expression from this strain was the highest among all strains we characterized (Fig. 7a–c and Supplementary Fig. 2, strain WP274). Performing an aging experiment using this strain verified our hypothesis by showing that both CV and the Fano factor were already at low levels even in young cells and they stayed low during the aging process (Fig. 7d–g). The flat nature of the YFP expression levels further indicated that the fraction of time the synthetic promoter was open did not decrease during aging (Fig. 7c). To bring further mechanistic support on these results, we applied our stochastic model to this strain. After extracting the values of *r*_ON_ and *r*_OFF_ rates in young cells using our model, we systematically varied these rates to mimick the impact of aging on them and calculated the resulting noise values (Supplementary Fig. 5). Once again, we saw that only certain trajectories of changes in *r*_ON_ and *r*_OFF_ were allowed (Supplementary Figs. 5–7) if noise and YFP expression levels were to display flat profiles as shown experimentally (Fig. 7), providing further support on our proposed mechanism that the noise dynamics during aging is largely governed by aging-associated changes in chromatin transition rates.

### Impact of non-chromatin reaction rates on noise

The rates of transcription and translation, and the stability of their product, may change during aging. To determine whether changes in those rates, without more, might account for the noise changes we observed, we computationally examined more than 1,600 sets of parameters in which all promoter-dynamics parameters were kept constant but the messenger RNA synthesis and degradation rates were changed by up to 10-fold in both directions from their original values, adjusting the translation rate as necessary to keep the expression level roughly constant. Although we did observe slight noise decreases in some cases (Supplementary Fig. 8), they were not enough to account for the level of noise decrease observed experimentally.

### Similar noise dynamics observed with the *HHF2* promoter

To rule out the possibility that the noise reduction phenotype observed in aging cells is a feature of the P_*GAL1*_ only and not a potentially general phenotype associatable to other promoters, we measured the activity of the *HHF2* promoter in aging cells. This promoter drives the expression of histone H4 gene whose expression products are core histone proteins required for chromatin assembly. Inserting a single copy of the P_*HHF2*_-YFP construct into yeast genome and following the stochastic fluctuations of YFP expression in aging cells, we observed a noise-reduction pattern (Supplementary Fig. 9) that was similar to the one observed from the P_*GAL1*_-YFP construct in the same wild type background (Fig. 2e–h). These results suggest that the noise-reduction behavior we have characterized is not a rare trait that is only attributable to the P_*GAL1*_ of the GAL network.

It is worth noting that even though *HHF2* is tightly regulated during the cell cycle, we did not observe massive intragenerational variations in YFP fluorescence intensity. We think this is due to several factors. First, the existing YFP reservoir in the cell will dampen the relative level of increase from a transcriptional burst. Moreover, *HHF2* is principally expressed during S phase, coinciding with budding and substantial cell volume growth. The resulting dilution can offset the impact of YFP production on the overall YFP concentration (which is what our experiments measured).

### No correlation between expression and cell division times

YFP is a stable reporter with cell division being the primary mechanism to remove it from a cell. To check that the gene expression variability measured during aging is not simply due to the variability in cell division times, we measured generation-specific correlations between single-cell YFP levels and cell division durations. We did not observe strong correlations between the two measures (Supplementary Fig. 10).

### Catastrophe during the last four generations of aging cells

We found the mean and the standard deviation of the generation durations to increase significantly within the final four generations of the mother cells tracked (Supplementary Fig. 11). While approaching their last generation, many cells displayed morphological changes and inefficiency in mother-daughter separation. Such phenotypes could be caused by increased rates of genome instability limiting proper cytokinesis. Indeed, a previous study^29^ demonstrated ~100-fold increase in loss of heterozygosity in old cells. To see how such catastrophic state in soon-to-die cells affects noise, separately for each strain characterized, we grouped the tracked mother cells with respect to their last generation and plotted the CV and Fano factor values (Supplementary Figs. 12–14). Noise levels increased significantly upon entry into the last four generations and they were the highest at the last generation, a trend similar to the earlier observations on the generation durations. Presumably, the abnormal variations observed in cell cycle durations during the last four generations of the mother cells and the increased noise levels are parallel realizations of the same global state of catastrophe occurring in dying cells. Cells lacking Rpd3 proteins displayed the lowest levels of increase in generation durations and noise during this state, indicating that they were relatively more protected from the catastrophic effects of the last four generations.

## Discussion

According to the previously proposed hypotheses and previous results based on animal studies, gene expression noise has been thought to increase during aging^24, 30–32^. In addition, uncontrolled increase in the expression of euchromatic genes has been proposed to occur during aging^24^. Here we characterize how aging alters noise in gene expression in single cells and propose a model composed of two distinct trends emerging by splitting the total lifespan of a cell into the ‘normal aging’ phase and the ‘catastrophe’ phase. We show that noise decreases during the normal aging phase and the observed noise reduction can be explained by increasing rates of chromatin state transitions, where the transition rates between the open and closed states of the chromatin are affected by aging in similar proportions and this can lead to steady gene expression profiles during aging, instead of increasing expression trends as proposed previously^24^. The catastrophe phase corresponds to the final four generations of a cell and we observe an increase in gene expression noise in this short phase.

Our hypothesis on the mechanism of noise reduction during the normal aging phase draws further support from the fact that aging is known to affect chromatin structure and remodeling in a number of ways. On a structural level, aging can cause substantial reorganizations of nucleosome and heterochromatin^33, 34^. On the histone level, it can cause different histone variants to be used and the abundance of the various histone modifications to change^33, 34^. Most pertinent here, aging has been shown to reduce the histone supply^24, 35^ and cause the formation of nucleosome-free patches in the chromatin^36^, which in turn is likely to cause increased transcriptional activation, consistent with our hypothesis that *r*_ON_ increases during aging. Our observation that introducing nucleosome disfavoring sequences into the constitutively active strain is sufficient to abolish the aging-related noise-reduction phenotype provides additional support for the idea that the chromatin structure at the promoter plays a crucial role in this process.

Although there are a number of technical and biological differences between the present work and the previous studies that might have contributed to the different conclusions, we note that the previous studies were by design incapable of observing the same cell in both young and aged state. Instead, completely distinct populations of cells were taken from younger and older animals, and their mRNA content was analyzed and compared. The inability to observe the same set of cells as they age significantly complicates the interpretation of the previously reported results and limits their value. Moreover, the previous studies all measured cell-to-cell variations in expression level by comparing the transcriptome of individual cells and the noise levels reported in such experiments necessarily included both extrinsic (variations due to cell-to-cell differences in other cellular components) and intrinsic noise (variations due to the inherent stochasticity of the gene expression process)^2^. In contrast, here we measure the expression level variations within the same cell over a short time window and report ‘cell-intrinsic’ noise. Our results indicate that intrinsic noise decreases as cells age, whereas the previous results, if accurate, suggest the total noise in a population of cells increases during aging. Thus, taken together, they suggest that the aging process may cause a reduction in intrinsic noise (before the catastrophe phase), but an increase in extrinsic noise.

The properties of a complex system are not always predictable by knowledge of the parts composing the system. Starting from the level of individual components, at each level of complexity, new rules can govern the system in ways that are not simply additive by summing up the information about those components. Such properties are defined as emergent properties^37, 38^. Fitting into this definition, genetic noise is a collective phenotype which cannot be determined based on an individual measurement. Results from this study suggest that noise reduction is an emergent property of single-cell aging when cells undergo normal aging in the non-catastrophic phase.

## Methods

### Construction of plasmids and yeast strains

All *S. cerevisiae* strains used in this study have the haploid BY genetic background. Complete descriptions of all strains are provided in Supplementary Table 1. The strain yTY10a carries the wild-type background and it has one copy of the P_*GAL1*_-YFP reporter construct integrated in its *ho* locus. For this, *Kpn*I*−P*_*GAL1*_ *−Bam*HI and *Bam*HI*−YFP−Eco*RI fragments were cloned into a plasmid upstream of the *CYC1* transcriptional terminator. The plasmid also carried the *P*_*TEF1*_-*HIS5* marker positioned to the left of the P_*GAL1*_-YFP construct. Using this plasmid as a template together with 5′- and 3′-primers homologous to the *ho* locus (Supplementary Fig. 15), the [P_*TEF1*_-*HIS5* + P_*GAL1*_-YFP] region of the plasmid was PCR amplified and then transformed into yeast. The P_*GAL1*_ sequence corresponds to the 669 base-pair region directly upstream of the start codon of the *S. cerevisiae GAL1* gene. Strains WP190 and GESC19 also have the same single-copy reporter construct integrated in their *ho* loci.

Carrying one copy of the P_*HHF2*_-YFP reporter in its *ho* locus, the strain WP268 was constructed by following the same cloning steps as described above, except that the wild-type *HHF2* promoter was used instead of the P_*GAL1*_. The P_*HHF2*_ promoter sequence corresponds to the full intergenic region directly upstream of the start codon of the *S. cerevisiae HHF2* gene.

For the deletion of the *GAL80* or *RPD3* gene in the relevant strains (Supplementary Table 1), *KanMX* or *CaURA3* cassette was amplified (using PCR) from plasmids carrying the cassette by using 5′- and 3′-primers homologous to the region immediately up- and downstream of the deleted gene (Supplementary Fig. 16). The transformed colonies were selected on plates missing uracil or containing the drug G418. The deletions were verified by PCR.

The strains GESC11 and WP274 carry one copy of the synthetic P_*GAL1∗*_-YFP reporter in their *ho* loci and they were constructed using the appropriate primers (Supplementary Fig. 17) and the same cloning steps as the ones used for the construction of the wild-type P_*GAL1*_-YFP reporter described above. In order to obtain the synthetic P_*GAL1∗*_ promoter sequence, we took the wild-type P_*GAL1*_ and inserted nucleosome-disfavoring sequences into it at three different locations. The full sequence of the synthetic promoter is shown in Supplementary Fig. 18.

### Preparation of the cells for the microfluidics experiments

Cells were grown in an incubator-shaker environment for 48 h before being transferred to the microfluidic chip. The goal of the shaker growth phase was to bring the YFP expression levels to steady state.

Cultures were grown in complete synthetic media supplemented with amino acids. Glucose (0.2%) and galactose (0.5%) were used as the carbon sources. These concentrations were chosen to provide appreciable but not saturating network induction, while ensuring that uninduced cells do not suffer a significant fitness disadvantage. Using 10 ml culture volumes in a shaker set to 30 °C, yeast cells were grown for 24 h and the YFP expression distributions were measured using a flow cytometer (FACSverse, Becton-Dickenson). The same cultures were diluted and put back into the shaker for the goal of taking another fluorescence-activated cell sorting (FACS) measurement at the 48 h time point. For the analysis of the flow cytometry data, a small gate was applied on the forward/side scattering plot. On average, ~8,000 cells fell into each gated area and their YFP fluorescence values were quantified. Fractions of ON cells were quantified using a cutoff separating the OFF and ON cells, and the mean fluorescence of the ON cells were calculated.

By diluting the starting cultures appropriately, the cell densities (OD_600_) at the times of FACS measurements were made to be around 0.1. All cultures were prepared and ran in independent duplicates. After the 48 h time point, cells were ready to be used in the microfluidic chip experiments as described below.

### Protocol for and analysis of the microfluidics experiments

The design and fabrication of the polydimethylsiloxane (PDMS) chip used in this study and the experimental protocols for setting up and running the aging experiments were provided in detail in our previous publication^15^. After growing cells for 48 h in the shaker environment as described above, cells were loaded into the microfluidic chip using a syringe pump operating at the flow rate of 20 μL min^−1^ for 3 min. The cell density (OD_600_) at the time of the loading was around 0.1. The type and composition of the minimal media used during the chip experiments were the same as the ones used in the shaker growth phase.

Using NIKON’s Elements software, mother cells were analyzed for their life span values by starting from their first generation until the end of their RLS. For tracking the cells, a ×60 oil objective was used. Bright-field and YFP images were acquired with 10 min intervals. The bright-field images were used to quantify the number of budding events and daughter production durations throughout the lifespan of each trapped mother cell. This way, single-cell life span values were quantified and recorded after the completion of each aging experiment. The bright-field images were started to be taken right after cell loading into the microfluidic platform, while YFP snapshots were started after 7 h. During the 7-h period, newborn virgin cells were tracked while they were being trapped and stabilized in the trapping units of the platform. Following this protocol was important for avoiding phototoxicity during the initial preparation phase of each aging experiment and it was also helpful for tracking the exact age of each cell.

During each aging experiment, the syringe pump was programmed to push fresh minimal media into the microfluidic chip at two different media flow rates: the continuous rate at 2 µL min^−1^ for 18 min, followed by the flushing flow rate of 30 µL min^−1^ for 2 min. These flow rates cycled repeatedly until the end of the RLS experiment. The growth temperature during the aging experiments was ~30 °C.

The time series YFP fluorescence images were analyzed using NIKON’s Elements software. They were analyzed frame by frame, with each frame corresponding to the snapshot of one of the recorded locations at each time point. In each frame, each trapped mother cell was circled using a fixed-size circle in order to get the average YFP intensity inside the circled area at that time point. The circle covered only the mother compartment, even if there was a bud attached to it. As the mother and daughter compartments share the same cytoplasmic content until mitosis, average YFP pixel intensity served as a robust metric not sensitive to which compartment was circled. Then, for each frame, the background fluorescence intensity was measured and subtracted from the average YFP intensity reading of each trapped cell, giving rise to the final single-cell YFP intensity values at each time point. To obtain the background fluorescence level of each separate frame imaged at each separate time point, the fluorescence intensity of all pixels in a frame were sorted from lowest to highest. As expected, a large portion of all pixels carried very low fluorescence values, leading to a long tail of very low fluorescence intensities when plotted after sorting. Finally, consistently for each frame, the intensity of the bottom 10%th pixel of each frame was identified as the background fluorescence of the frame under analysis. The bottom 10% pixel intensities were part of the long tail of very low fluorescence intensities obtained after sorting all fluorescence intensities of the frame under analysis. Only ON-state cells were included in subsequent analysis, excluding OFF cells. An OFF cell was defined as a cell whose average YFP intensity throughout its lifespan was below 1,000 (arbitrary units).

The bright-field images facilitated the association of the multiple YFP snapshots taken of each mother cell during each generation to its specific generation number. Using non-overlapping windows with a size of two consecutive generations, for each mother cell separately, the YFP snapshots falling into each such window (at least eight snapshots over two generations of a mother cell) were used to obtain the CV, Fano factor, or average YFP value for the mother cell under analysis. Then, these window-specific single-cell values were averaged across all cells that went through the same two-generation-long window, resulting in the plots for the CV, Fano factor, and mean YFP. In order to avoid the confounding effects of the catastrophe phase of each cell (its last four generations), the analyzed/plotted data did not include the data taken during the last four generations of each cell (except the data of Supplementary Figs. 1 and 11–14). Furthermore, cells living <10 generations were not included in this study, except in Supplementary Fig. 1 where such short-living cells were still kept for the construction of the full cellular viability curves and for the quantification of the average lifespan values for each strain. Although the number of such short-living cells were low among all cells for each strain, we still opted not to include them in the study so that confounding effects due to potential cell sickness would be avoided.

### Code availability

The code implementing the stochastic model is available from the corresponding author upon request.

### Data availability

The data that support the conclusions of this study are available from the corresponding author upon request.

## Electronic supplementary material

Supplementary Information
