# Supplementary material for: Noise reduction as an emergent property of single-cell aging
Source: Nat Commun. 2017 Sep 25;8:680. doi: 10.1038/s41467-017-00752-9 (PMC5613028; doi:10.1038/s41467-017-00752-9)
Supplement: Supplementary file 1 — Supplementary Information [file 41467_2017_752_MOESM1_ESM.pdf]

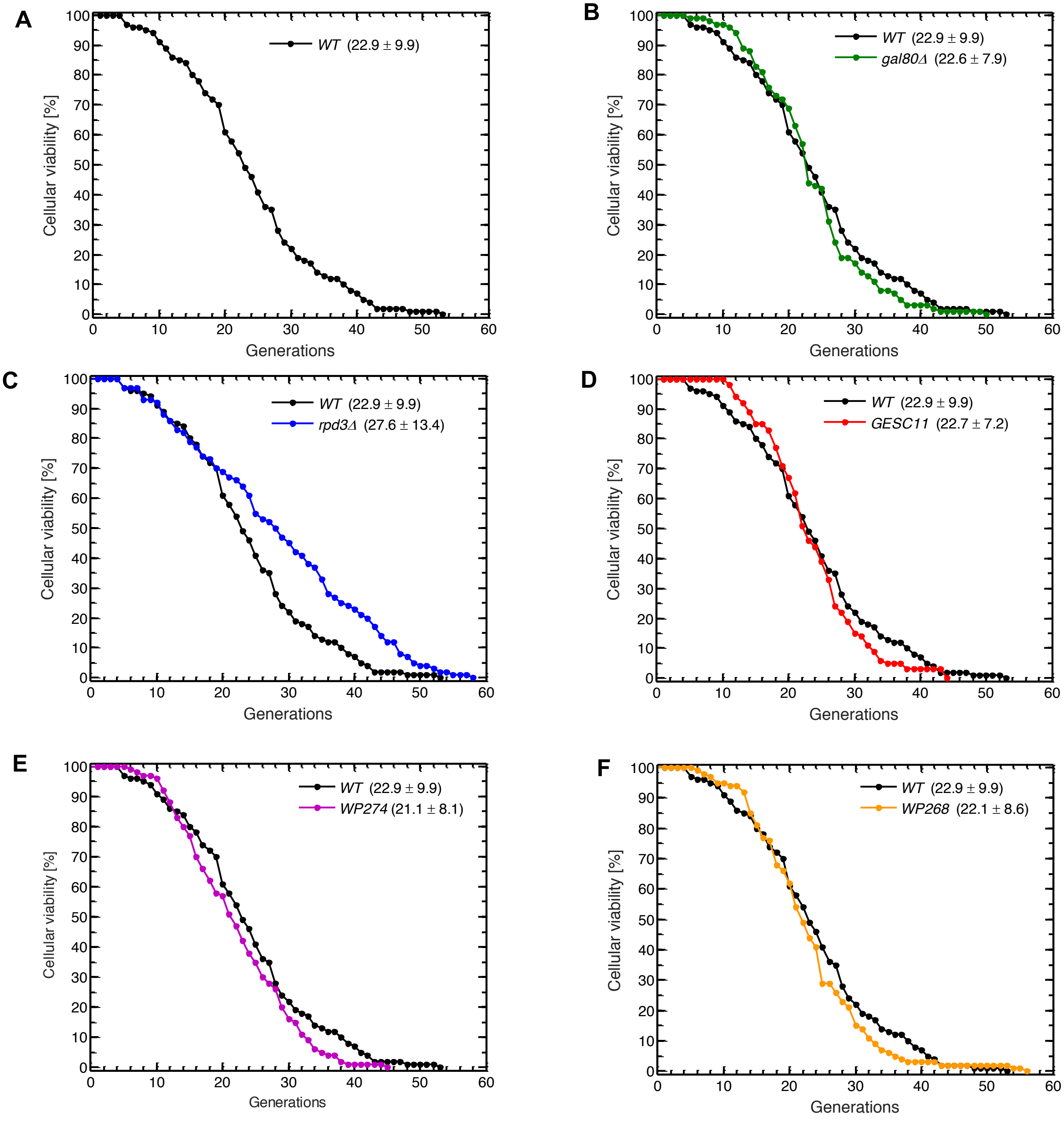

Supplementary Fig. 1. (**A**) Cellular viability curve for WT strain (black dot and solid line), N=100 cells are studied. (**B-F**) Cellular viability curves for *gal80Δ* (**B**), *rpd3Δ* (**C**), GESC11 (**D**), WP274 (**E**), and WP268 (**F**) respectively, in comparison with the curve for WT strain (black). N = 100 cells are used to construct each curve.

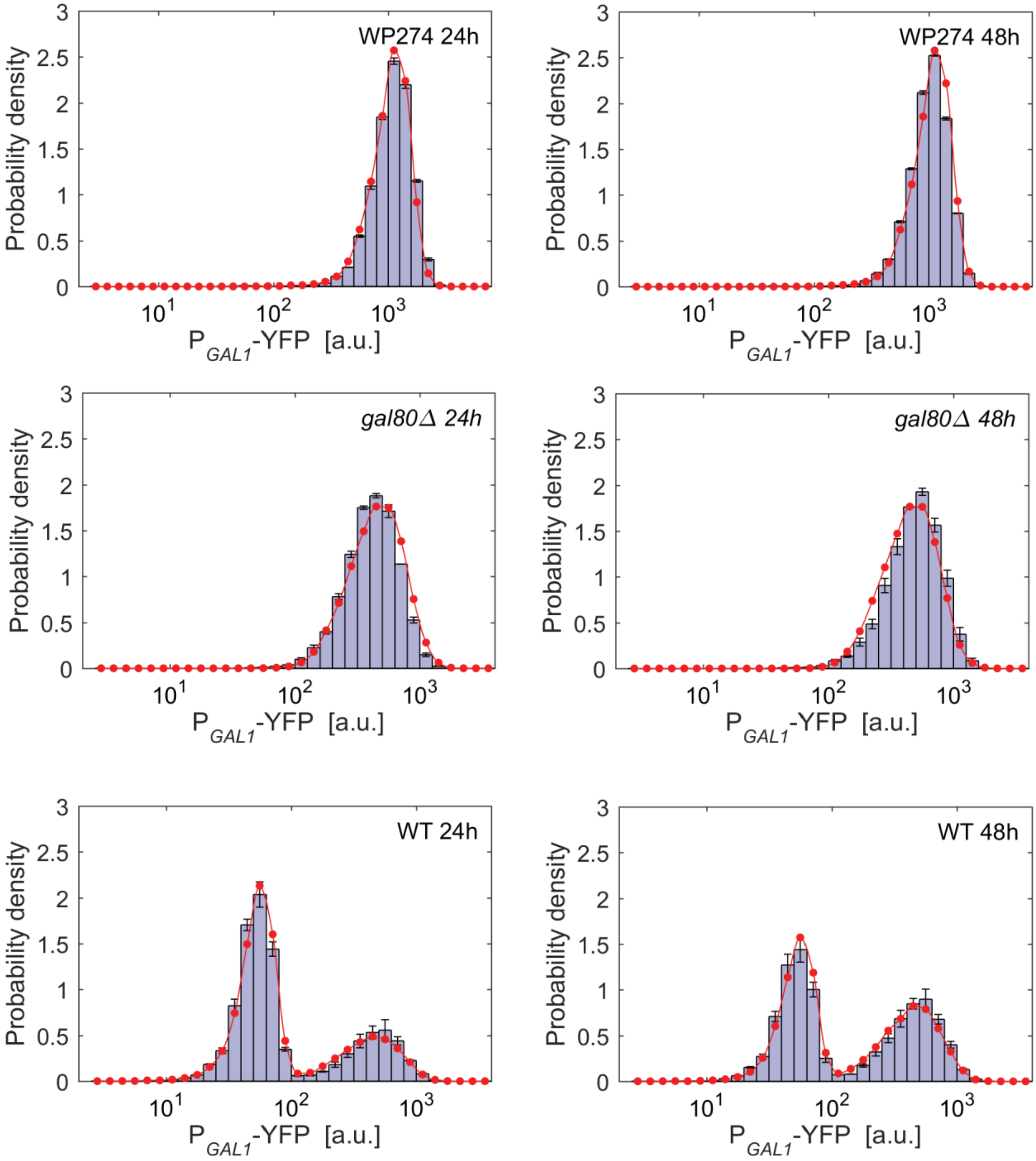
WP274 24h
WP274 48h
gal80Δ 24h
gal80Δ 48h
WT 24h
WT 48h
Probability density
P GAL1 -YFP [a.u.]
0
0.5
1
1.5
2
2.5
3
10^1
10^2
10^3

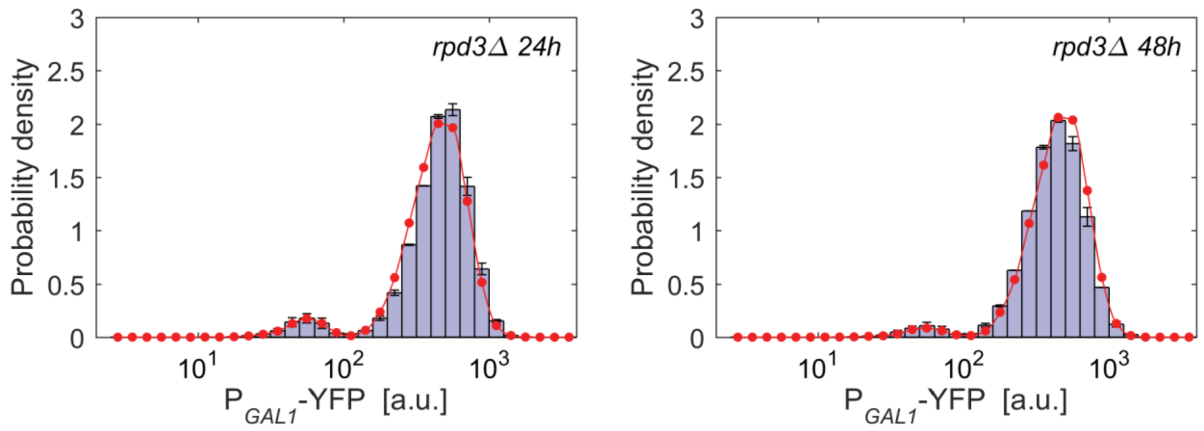

Supplementary Fig. 2. Comparison of fitting results (red) and experimental data from flow cytometry (bars) for all four strains (WP274, *gal80Δ*, WT, *rpd3Δ*). Error bars indicate SEM (N=2).

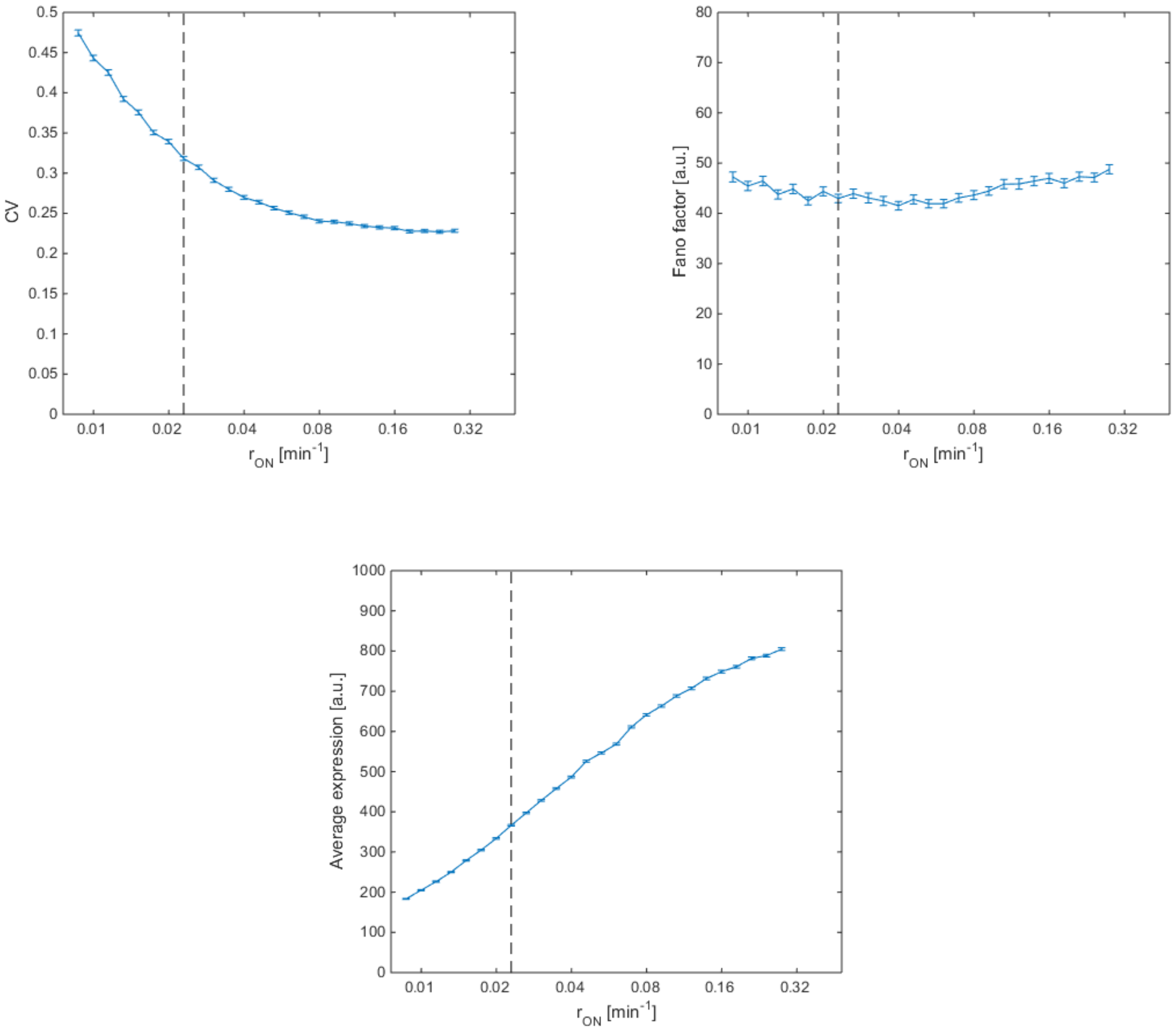

Supplementary Fig. 3. Effect of r_{ON} on noise in expression (measured by CV and Fano factor) and average expression level. For these simulations, the value of r_{OFF} is fixed at the fitted value for the native GAL1 promoter (0.0348 min^{-1}). The dashed line indicates the fitted r_{ON} value for the native GAL1 promoter (0.0229 min^{-1}). Error bars indicate SEM (N=2000).

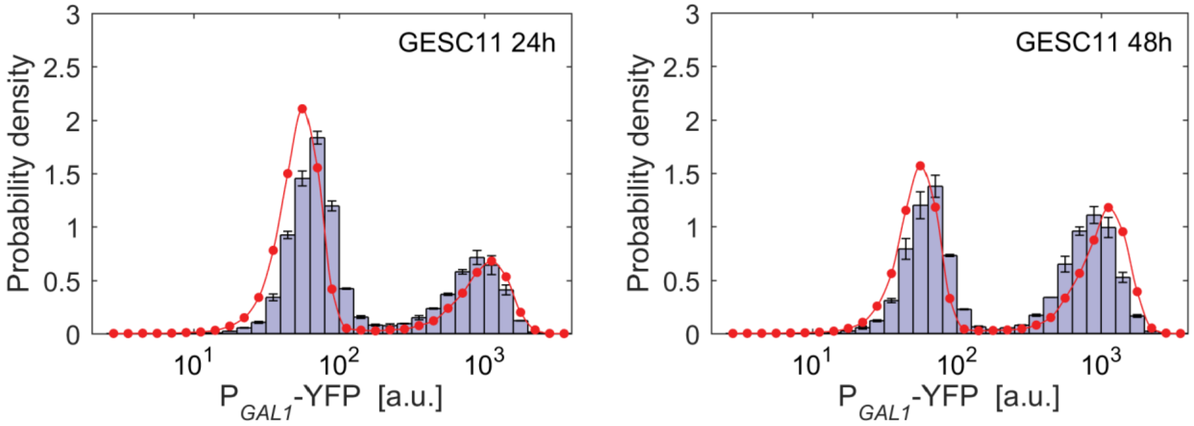

Supplementary Fig. 4. Comparison of model prediction (red) and experimental data from flow cytometry (bars) for the strain GESC11. Error bars indicate SEM (N=2).

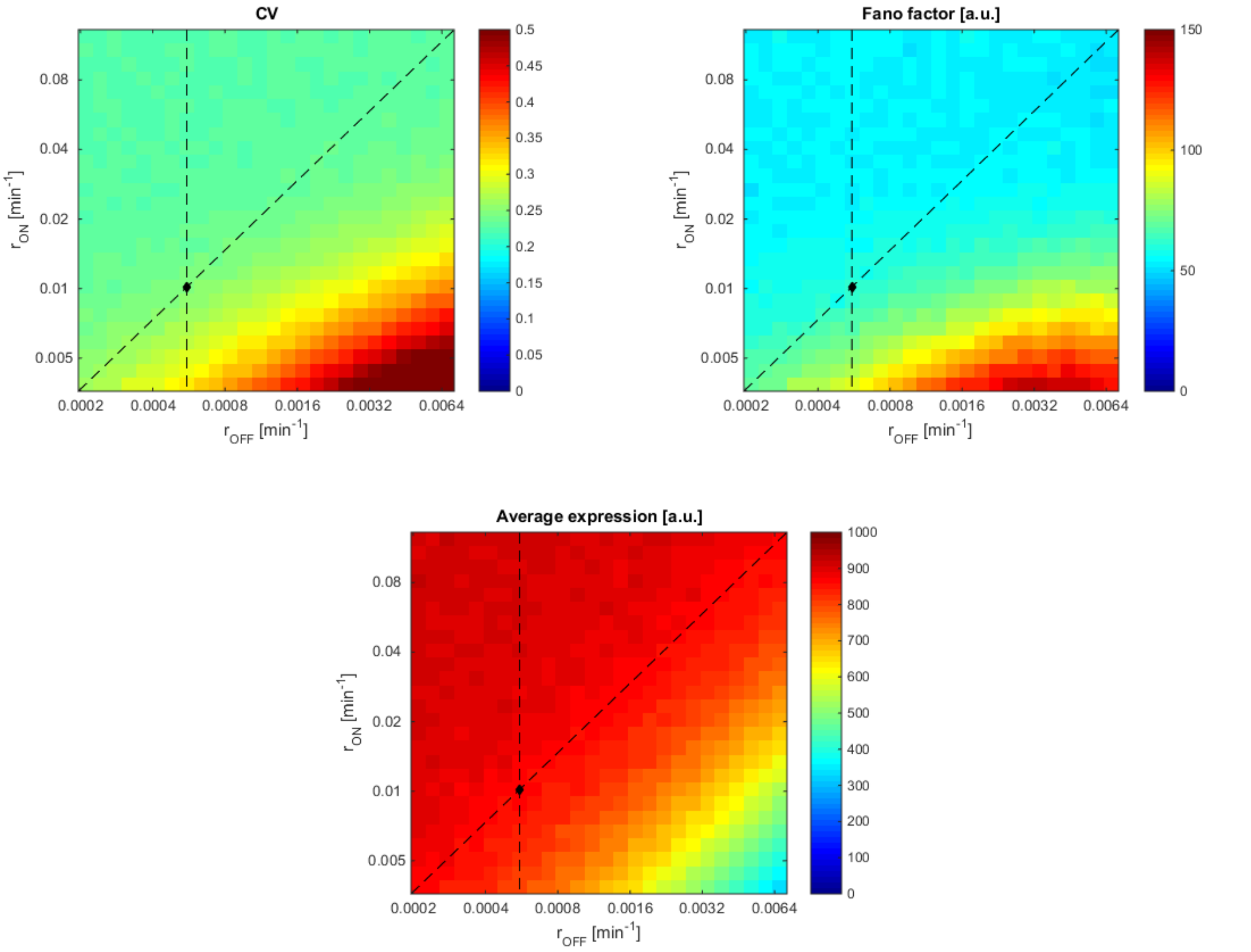

Supplementary Fig. 5. Effect of r_{ON} and r_{OFF} on noise in expression (measured by CV and Fano factor) and average expression level. The black diamond indicates the fitted parameter values of the modified GAL1 promoter (r_{OFF} = 0.00055 min^{-1}, r_{ON} = 0.0102 min^{-1}). The vertical dashed line indicates the range of parameter values used for the simulations in Supplementary Fig. 6, where r_{OFF} is kept constant. The diagonal dashed line indicates the range of parameter values used for the simulations in Supplementary Fig. 7, where the average expression level is kept constant by varying the value of r_{OFF} correspondingly.

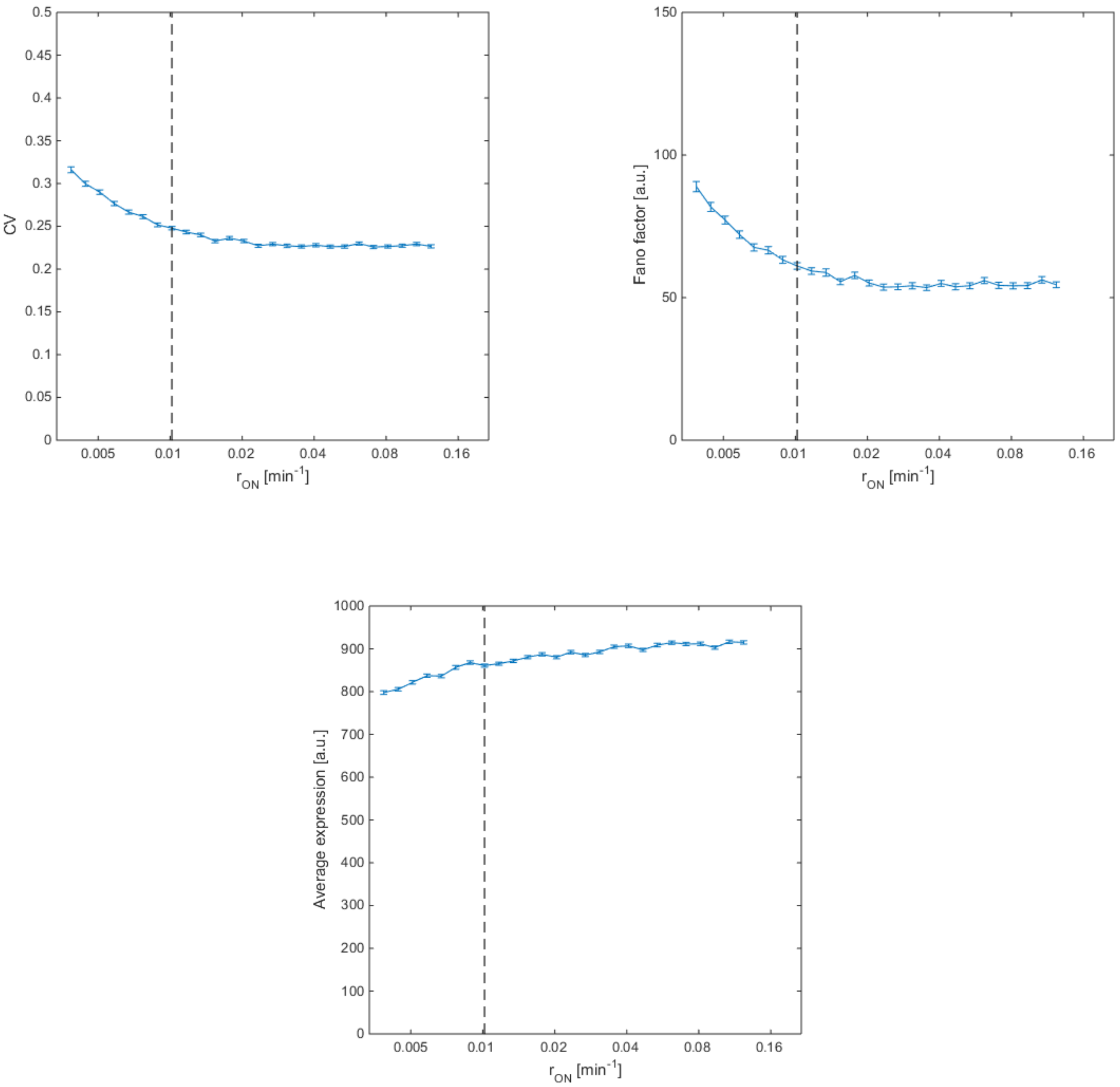

Supplementary Fig. 6. Effect of r_{ON} on noise in expression (measured by CV and Fano factor) and average expression level. For these simulations, the value of r_{OFF} is fixed at the fitted value for the modified GAL1 promoter (0.00055 min^{-1}). The dashed line indicates the fitted r_{ON} value for the modified GAL1 promoter (0.0102 min^{-1}). Error bars indicate SEM (N=2000).

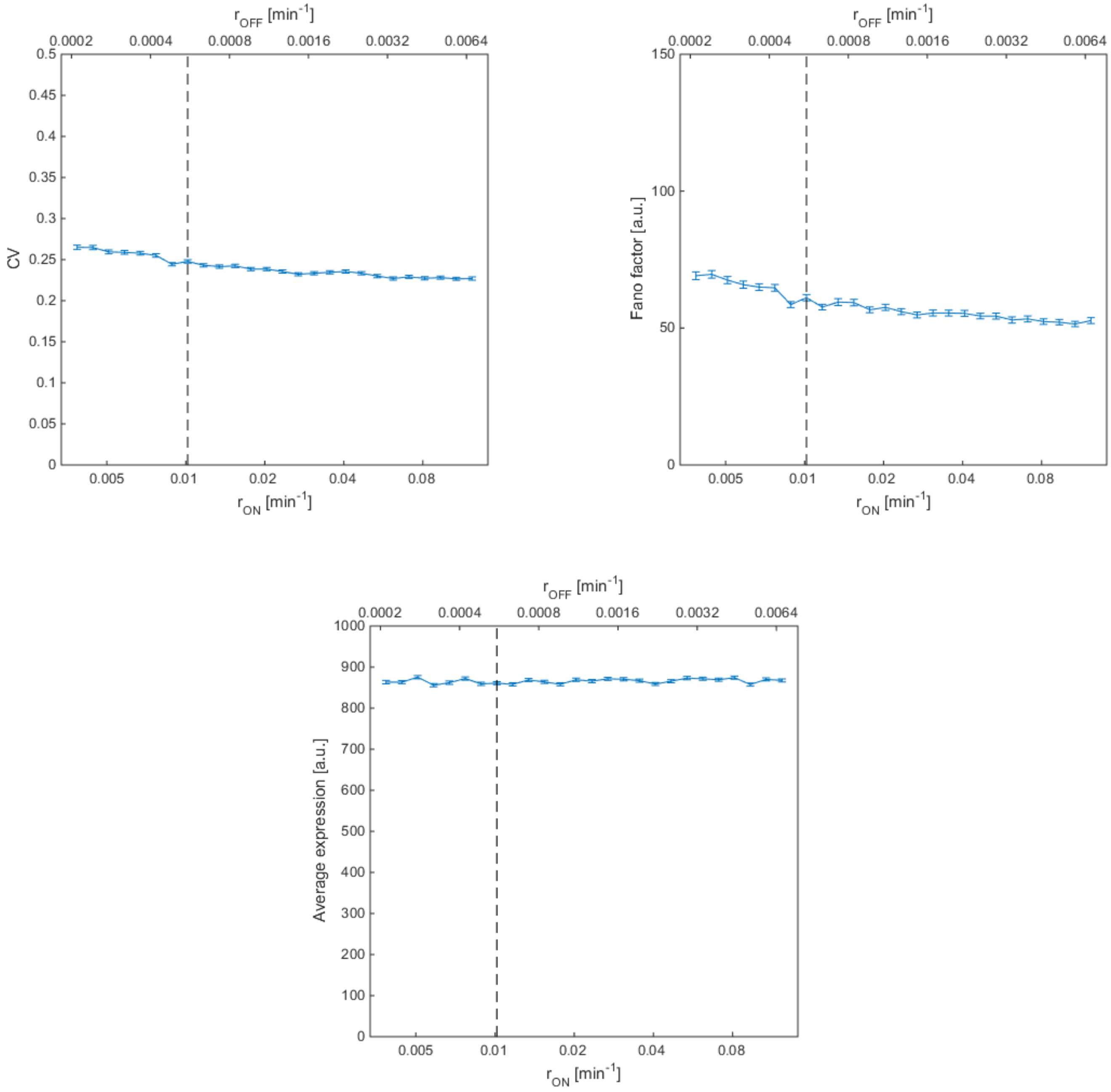

Supplementary Fig. 7. Effect of r_{ON} and r_{OFF} on noise in expression (measured by CV and Fano factor) and average expression level, where the average expression level is kept approximately constant by varying the two parameters at the same ratio. The dashed line indicates the fitted parameter values of the modified GAL1 promoter (r_{OFF} = 0.00055 min^{-1}, r_{ON} = 0.0102 min^{-1}). Error bars indicate SEM (N=2000).

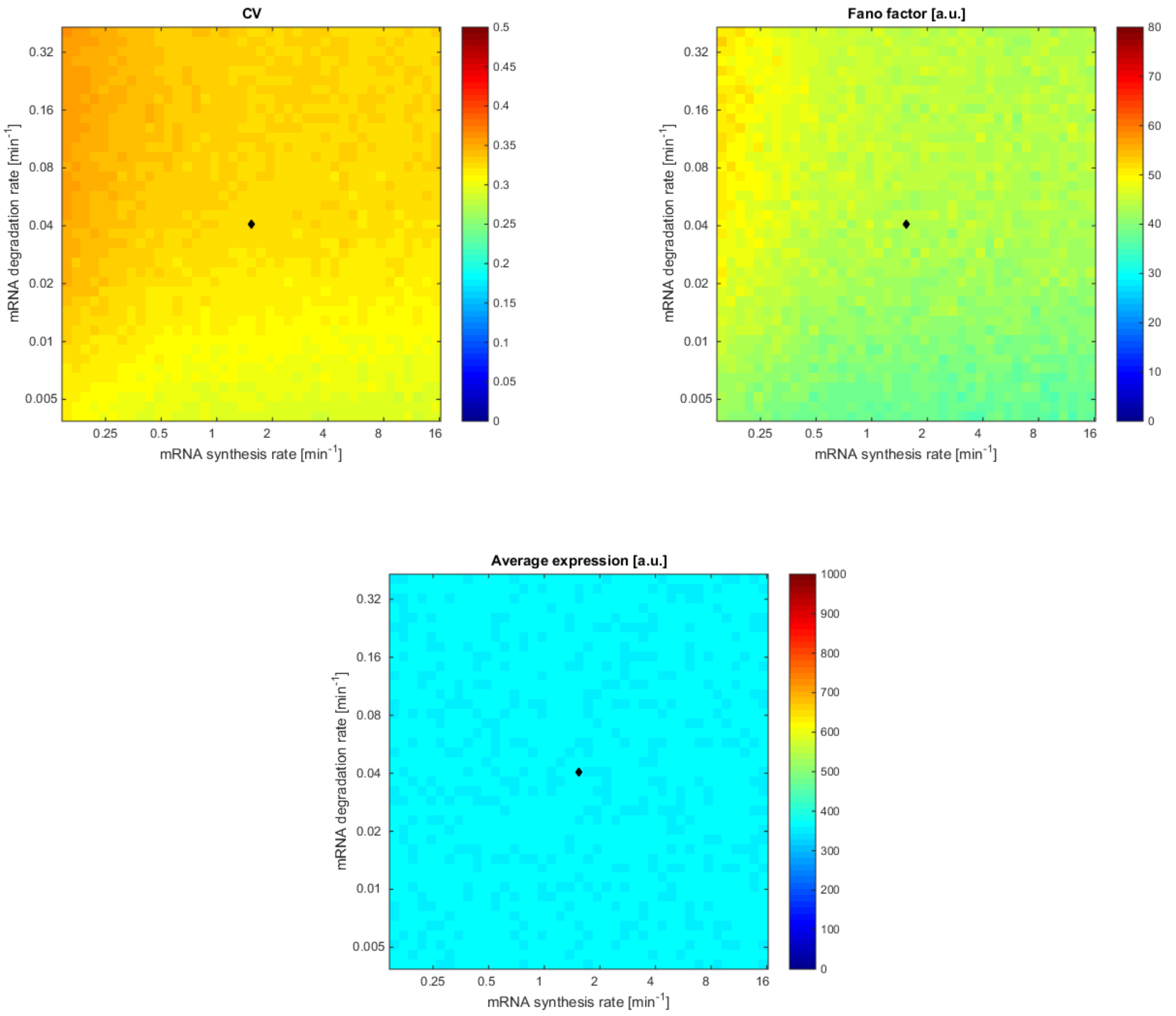

Supplementary Fig. 8. Effect of changing transcription, translation, and mRNA degradation rates on noise in expression (measured by CV and Fano factor), where average expression level is kept approximately constant by adjusting the translation rate constant. The black diamond indicates the synthesis and degradation rates for the mRNA used when originally fitting the model (synthesis rate = 1.5383 min^{-1}, degradation rate = 0.04077 min^{-1}; see Supplementary Table 3).

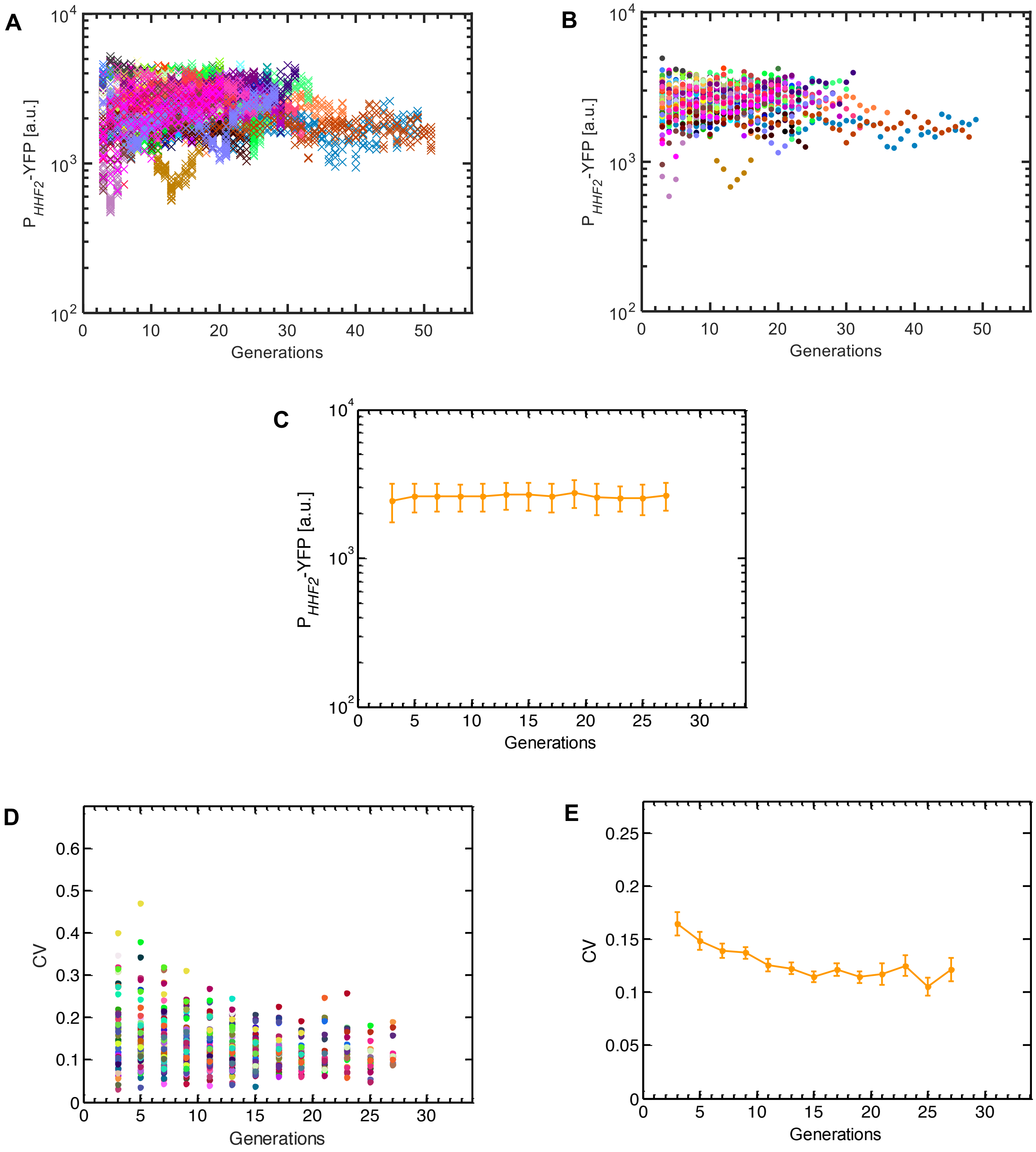
A
B
C
D
E
P_HHF2-YFP [a.u.]
Generations
CV

F

G

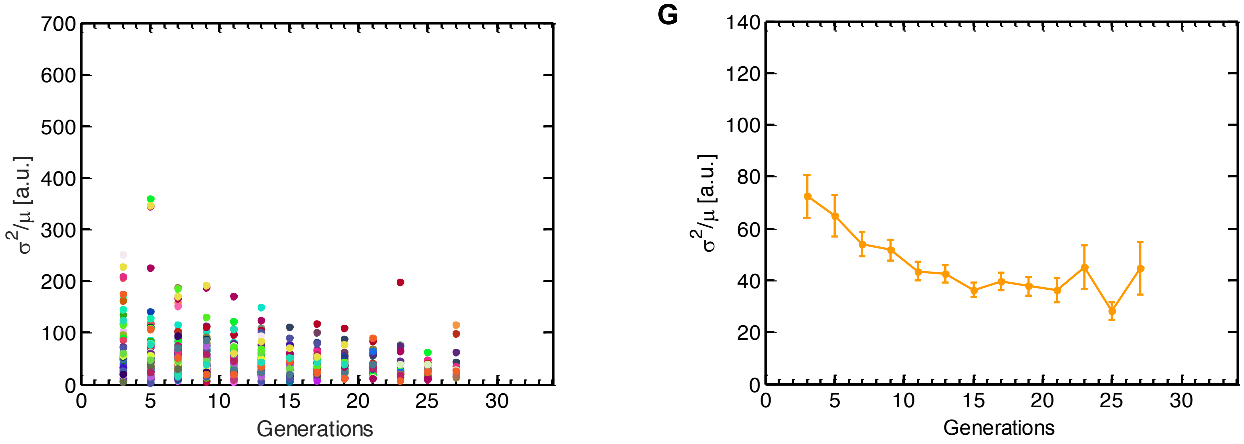

Supplementary Fig. 9. (**A**) Generational fluorescence levels for N = 82 ON mother cells of WP268 strain. Each generation has 4 measurements or more. All the mother cells used in the study have replicative lifespan of 10 generations or more. (**B**) Mean generational fluorescence levels for the cells described in (A). (**C**) The aging axis is divided into non-overlapping windows of two generations, and the mean and standard deviation (SD) of fluorescence levels across all available cells inside each window are plotted. The error bars denote SD, the number of data points used for the SD quantification are 10 or above. (**D**) CV values of individual cells inside each window. (**E**) Mean and SEM of the CV's across the cell population as shown in (D). (**F**) Fano factor values of individual cells inside each window. (**G**) Mean and SEM of the Fano factors across the cell population as shown in (F). For the SEM quantifications in (E,G), the number of data points used is at least 10.

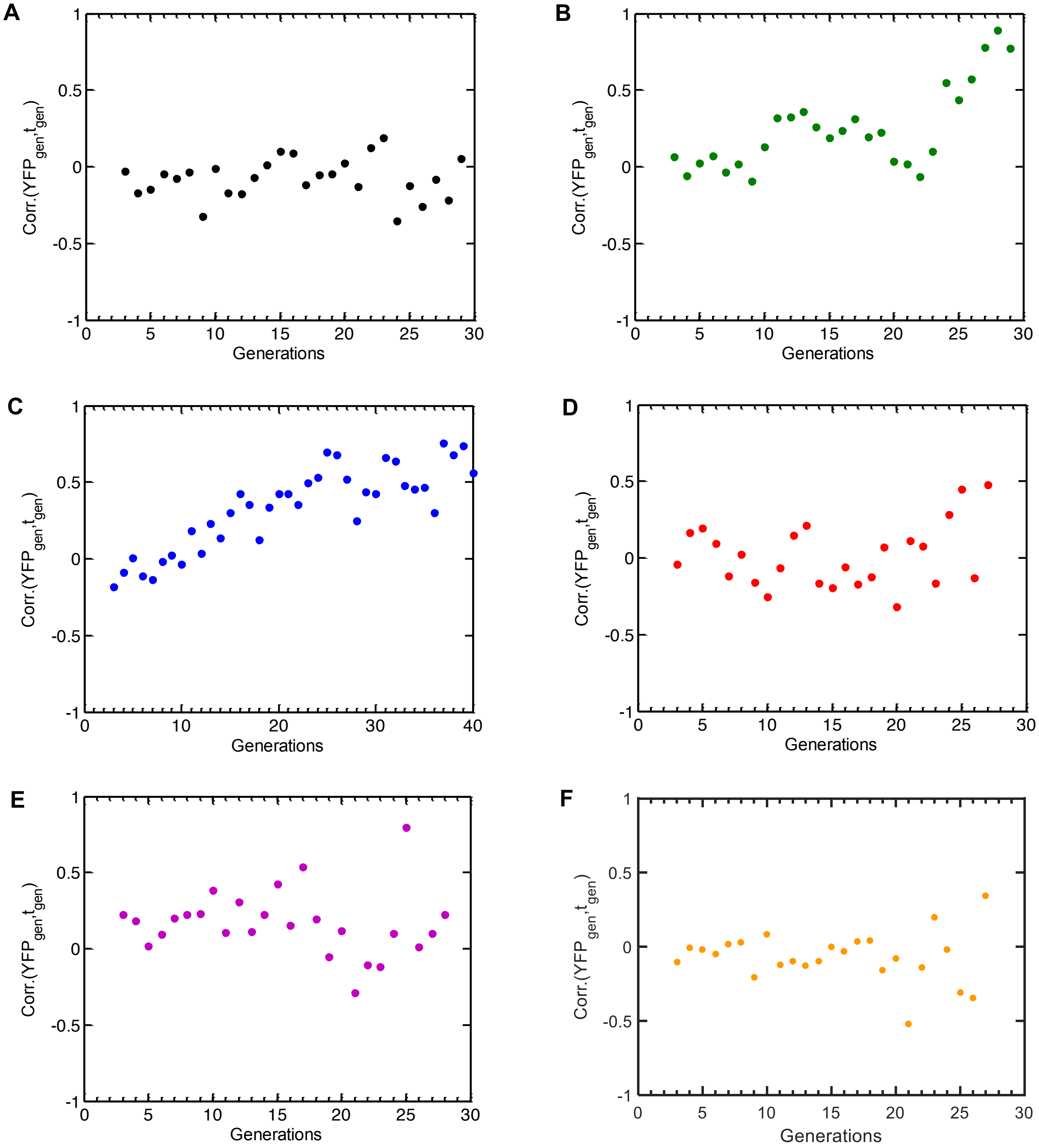

Supplementary Fig. 10. Pearson Correlation between generational fluorescence level and generation time for WT (**A**), *gal80Δ* (**B**), *rpd3Δ* (**C**), GESC11 (**D**), WP274 (**E**), and WP268 (**F**) strains respectively.

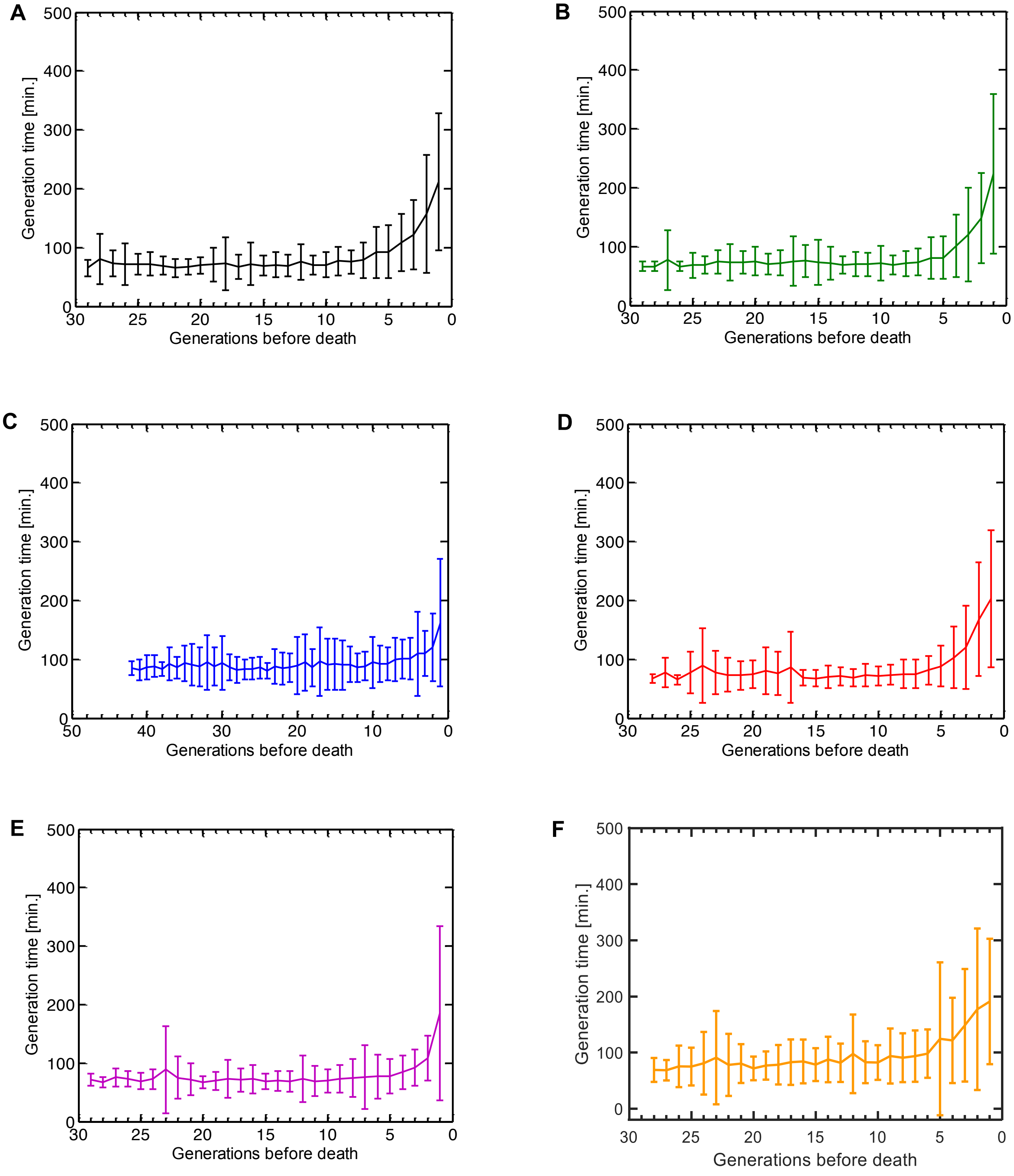

Supplementary Fig. 11. Mean population level generation time towards the death event for WT (**A**), *gal80Δ* (**B**), *rpd3Δ* (**C**), GESC11 (**D**), WP274 (**E**), and WP268 (**F**) strains respectively. Error bars denote SD with N = 10 or above.

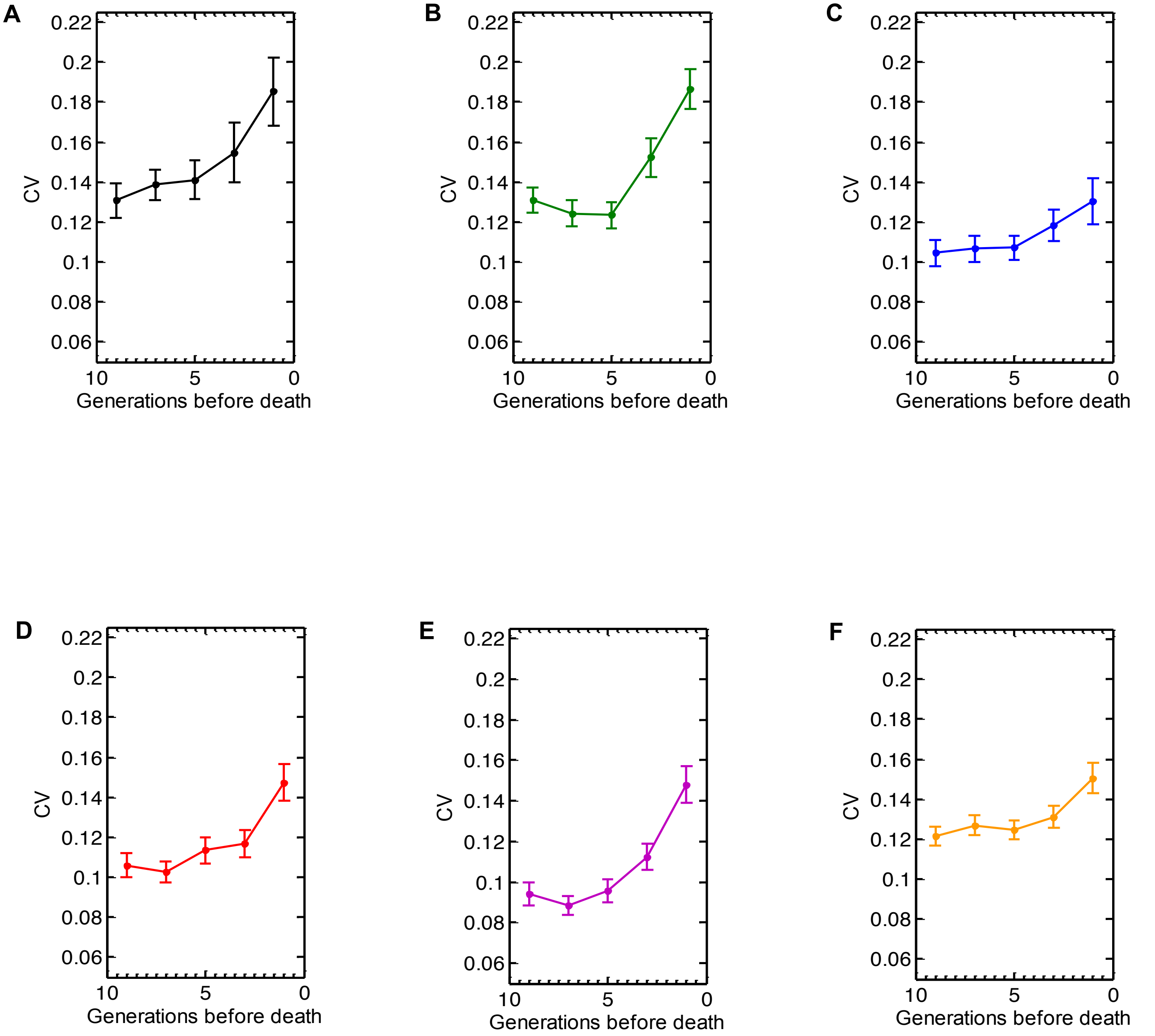

Supplementary Fig. 12. Mean single-cell level CV inside each window (window size = 2 generations) towards the death event for WT (**A**), *gal80Δ* (**B**), *rpd3Δ* (**C**), GESC11 (**D**), WP274 (**E**), and WP268 (**F**) strains respectively. Inside each window, CV value is computed for each available cell first (with minimum 8 fluorescence measurements) and then averaged across the cell population to get the population mean. Error bars denote SEM with N = 10 or above data points.

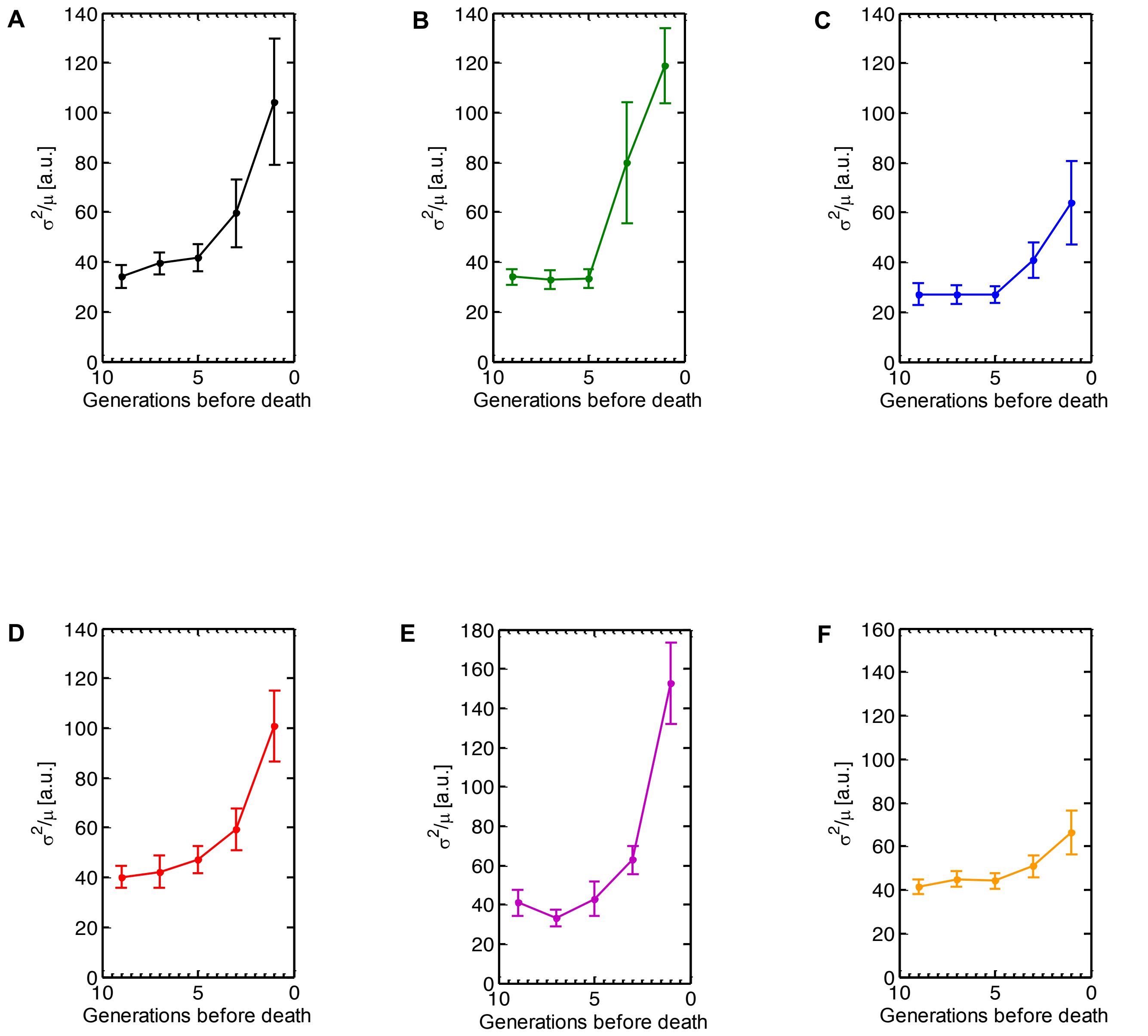

Supplementary Fig. 13. Mean single-cell level Fano factor inside each window (window size = 2 generations) towards the death event for WT (**A**), *gal80Δ* (**B**), *rpd3Δ* (**C**), GESC11 (**D**), WP274 (**E**), and WP268 (**F**) strains respectively. Inside each window, Fano factor is computed for each available cell first (with minimum 8 fluorescence measurements) and then averaged across the cell population to get the population mean. Error bars denote SEM with N = 10 or above data points.

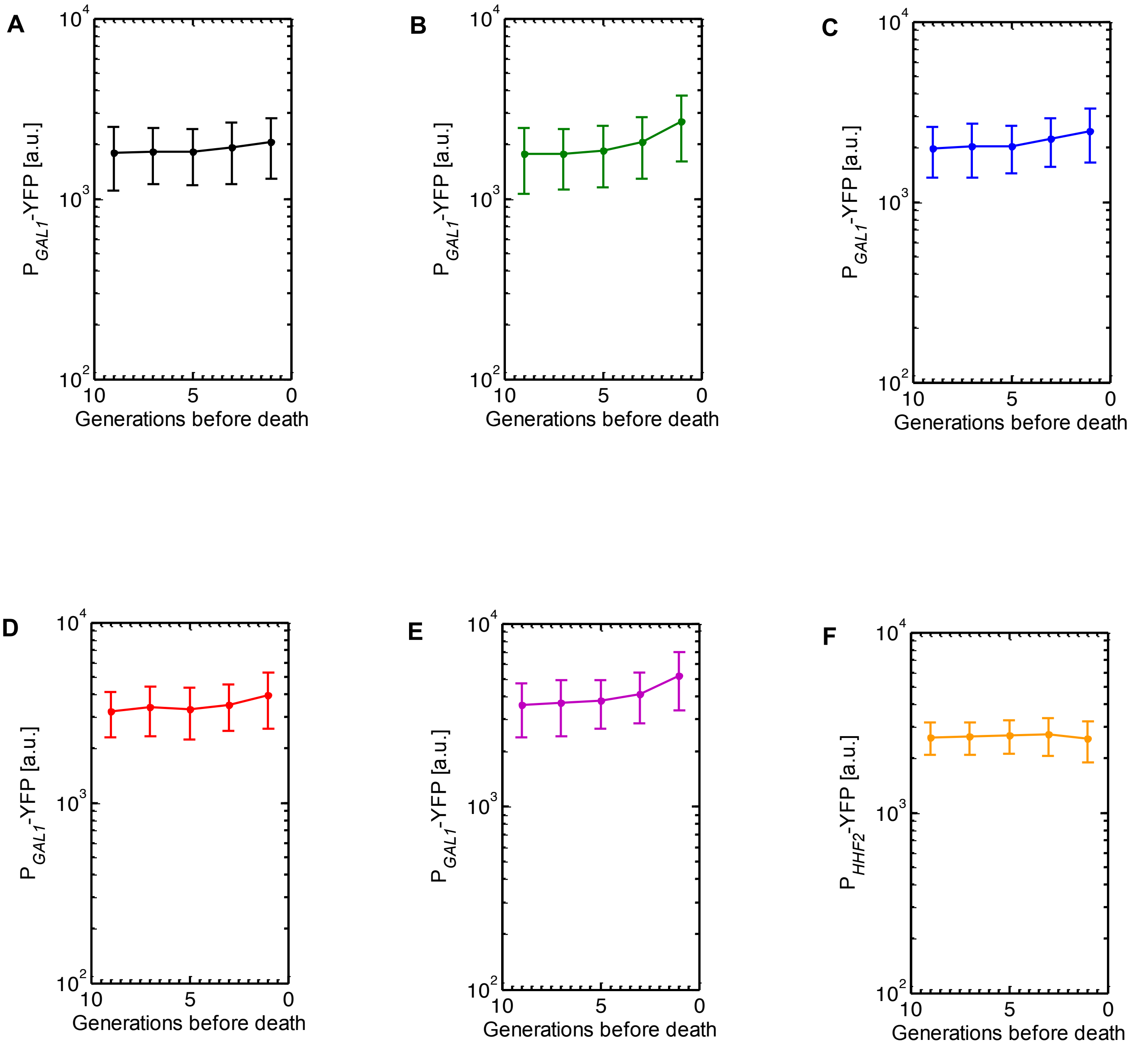

Supplementary Fig. 14. Mean fluorescence level inside each window (window size = 2 generations) towards the death event for WT (**A**), *gal80Δ* (**B**), *rpd3Δ* (**C**), GESC11 (**D**), WP274 (**E**), and WP268 (**F**) strains respectively. Inside each window, mean fluorescence level is computed for each available cell first (with minimum 8 fluorescence measurements) and then averaged across the cell population to get the population mean. Error bars denote SEM, with N = 10 or above data points.

Forward (5'):
TATTTGATCTTTACCGTTTAGTTCCAACGTAAAATTGTGCCTTTGGACTTAAAATGGCGTGCG
TTTCGGTGATGACGGT

Reverse (3'):
CTCTTGTTTTGTGACCACTTCGACAATATGACAAAACATTCTGTGAAGTTGTTCCCCCAGAC
CATGATTACGCCAAGCGC

Supplementary Fig. 15. Sequence of primers used for integration of P_{GAL1}-YFP into the *ho* locus.

***GAL80* Forward:**
GCTGGTCCTTGCCGACCAGCGTATACAATCTCGATAGTTGGTTTCCCGTTCTTTCCACTCCC
GTCAGCTTGCCTTGTCCCCGCCG

***GAL80* Reverse:**
CTCAGTATTCGTTTTTATAACGTTCGCTGCACTGGGGGCCAAGCACAGGGCAAGATGCTTG
AGCTCGATTACAACAGGTGTTGTCC

***RPD3* Forward:**
CTTAAGTGCCTTTTATTCACTTTTCTTCTTTTGTTTCACATTATTTATATTCGTATATACTTCCA
ACTCTTTTTTGCTAGGGATAACAGGGTAAT

***RPD3* Reverse:**
CCAAGCGATAAAAGACGCGTTGCATATTTTTACGATGCAGACGTTGGGAACTATGCATATGG
AGCAGGTCACCCGGTGGATCTGATATCATCGA

Supplementary Fig. 16. Sequence of primers used for deletion of the *GAL80* and *RPD3* genes.

Forward (5'):
TATTTGATCTTTACCGTTTAGTTCCAACGTAAAATTGTGCCTTTGGACTTAAAATGGCGT

Reverse (3'):
CTCTTGTTTTGTGACCACTTCGACAATATGACAAAACATTCTGTGAAGTTGTTCCCCCAG

Supplementary Fig. 17. Sequence of primers used for integration of P_{GAL1*}-YFP into the *ho* locus.

ttatattgaattttcaaaaattcttactttttttttggatggacgcaaagaagtttaataatcatattacatggcattaccaccatatacatatccatat ctaatcttacttatatgttgtggaaatgtaaagagccccattatcttagcctaaaaaaccttctctttggaactttcagtTTTTTTTTTCAT TTTTTTTTTTtatattgaagtaCGGATTAGAAGCCGCCGagCGGGCGACAGCCCTCCGaCGGAAGAC TCTCCTCCGtgcgtcctcgtcttcaccggtcgcgttcctgaaacgcagatgtgcCTCGCGCCGCACTGCTCCGAACA ATaaagTTTTTTTTTCATTTTTTTTTTTggttatgaagaggaaaaattggcagtaacctggccccacaaaccttcaaattaa cgaatcaaattaacaaccataggatgataatgcgattagttttttagccttatttctggggtaattaatcTTTTTTTTTCATTTTTTTTT TTtattaacagataTATAAAtggaaaagctgcataaccactttaactaatactttcaacattttcagtttgtattacttcttattcaaatgtcata aaagtatcaacaaaaaaattgttaatatacctctatactttaacgtcaaggagaaaaaactata

Supplementary Fig. 18. Sequence of the synthetic promoter, with the blue color showing the nucleosome-disfavoring sequences, the red color showing the binding sites for the Gal4 proteins, and the yellow color showing the location of the TATA box.

SUPPLEMENTARY NOTE 1: MODEL DESCRIPTION

We used a modified version of the stochastic model previously described[1] to model the GAL network genes (Supplementary Fig. 19). Briefly, each gene in model is modeled through a set of stochastic reactions: each gene has a promoter that switches between ON and OFF states, with full-speed transcription in ON state, and basal transcription in the OFF state. mRNA produced from transcription are translated into protein and both mRNA and protein can be degraded. The rate of switching from OFF to ON state is determined by the equation $r = r_{ON}F^{\eta}$, where r_{ON} is a model parameter (the maximum OFF-to-ON transition rate), η is a per-promoter nonlinearity coefficient representing the number of GAL4 binding sites in the promoter (1 for GAL3 and GAL80; 4 for GAL1), and F is a function representing the activity of the gene network, with the form

$$F = \frac{1}{1 + \left(\frac{S_{80}x_{80}}{1 + (S_3x_3g + S_1x_1g)^{\alpha}}\right)^{\beta}}$$

where S_1, S_3, and S_{80} are the scales of action of Gal1p, Gal3p and Gal80p, respectively, x_1, x_3 and x_{80} are the concentrations of Gal1p, Gal3p, and Gal80p, respectively, g is the inducer (galactose) concentration, α = 1 is the nonlinearity of Gal80p-Gal1p/Gal3p interaction, and β is the nonlinearity of Gal80p-Gal4p interaction. As in the previous work, we fixed S_{80} at the arbitrary value 30000 without loss of generality.

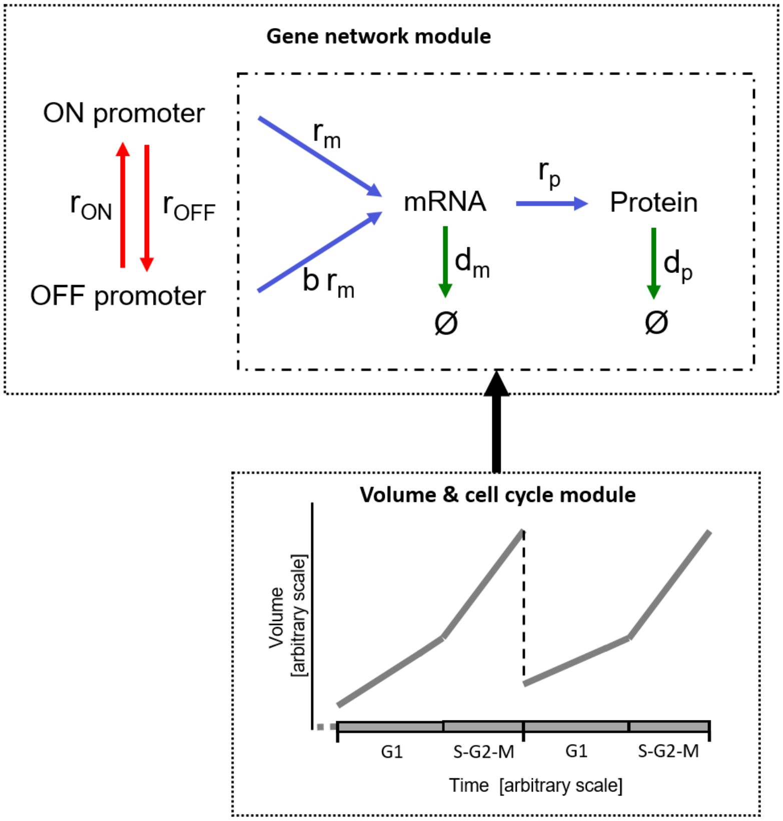

Supplementary Figure 19. Overview of the stochastic model. The model, as previously described[1], consists of two parts: a volume and cell cycle module based on previous work[2] and a gene network module consists of seven stochastic reactions (arrows) per gene (one gene shown). The rate constants for each reaction is shown next to the corresponding arrow. In addition to its indirect effects, the current volume directly affects the rate of five reactions (thick black arrow).

A separate volume model based on previous work[2] controls the cell cycle progression and volume dynamics. In this model, cells grow linearly at different rates in G1 and in S/G2/M, and the volume at which cells enter S is dependent on its G1 phase growth rate. Most volume growth in the mother compartment happens in G1, while the volume growth in S/G2/M predominantly goes to the daughter compartment. There is a lower bound on the time spent in G1 so that large cells do not immediately reenter S after M.

The volume model in previous work, however, is based on observations of predominantly young cells, and results in unrealistically large volumes when cells are allowed to grow and divide for many generations. To fix this problem, we decided to cap G1 phase volume at twice the volume of first-generation mother cells. Once the cap is reached, the cell volume no longer increases in G1, though the duration of G1 is not affected.

Volume model parameters (Supplementary Table 2) are obtained by scaling the parameter values in Ferrezuelo *et al.* to a doubling time of 100 minutes. All translation, transcription and degradation rates as well as the parameters of the volume model were fixed from literature values where available, and from ranges reported in the literature otherwise (Supplementary Table 3).

The remaining parameters are determined as follows:

- First, the promoter dynamics parameters (r_{ON}, the maximum OFF-to-ON transition rate, and r_{OFF}, the ON-to-OFF transition rate) for the modified GAL1 promoter, as well as the YFP-to-fluorescence conversion factor were determined (Supplementary Table 4) by fitting to data obtained from the WP274 strain, where the reporter is constitutively expressed.
- Second, the promoter dynamics parameters (r_{ON}, the maximum OFF-to-ON transition rate, and r_{OFF} , the ON-to-OFF transition rate) for the wild-type GAL1 promoter were determined (Supplementary Table 6-7) by fitting to data obtained from the *gal80Δ* strain.
- Third, the promoter dynamics parameters of the GAL3 and GAL80 promoters, along with the scales of action of GAL1 and GAL3 proteins and the nonlinearity of GAL4-GAL80 interaction (S_1, S_3 and β above), were determined (Supplementary Table 8) by fitting to data obtained from the wild-type strain, using the parameter values determined in the previous steps.
- Finally, the promoter dynamics parameters in the *rpd3Δ* strain was determined (Supplementary Tables 9-10) by fitting to data obtained from that strain, keeping all other parameter values fixed to those obtained in the previous steps.

In each step of the fitting procedure above, certain other model parameters are either fixed beforehand based on experimental observations, or calculated during the fit using the fitted parameters. These parameters are listed in Supplementary Tables 5, 7 and 10.

In each case, experimental data was obtained by flow cytometry after 24 and 48 hours of induction in CSM media containing 0.5% galactose and 0.2% glucose, and the model was fitted to the data in a manner similar to that previously described[1] to obtain the parameter values. Briefly, for every set of parameters, a population of 25000 cells were simulated for 24 hours and 48 hours, respectively, with resampling every 40 minutes to keep the population size roughly constant; at the end, the YFP protein level in each cell in the population was calculated and converted to simulated fluorescence level and aggregated to produce a simulated fluorescence distribution. The well-known Nelder-Mead optimization algorithm[3], as implemented in the NLopt library[4], is used to find the set of parameters that maximize the likelihood of observing the experimental fluorescence distribution.

We used the model to predict the expression histogram of the GESC11 strain (without further fitting) by taking the parameter values obtained from the WT strains, and substituting the parameter values for the reporter from the WP274 strain. The results are shown in Supplementary Fig. 4.

To determine the effects of the promoter dynamics parameters r_{ON} and r_{OFF} on the level of noise, we systematically varied the value of each in a wide range (between 38% and 1200% of the fitted value) in the *gal80Δ* model, keeping all other parameters constant, and evaluated their effects. For this analysis, we simulated a population of 20,000 exponentially growing cells that was induced for 48 hours, with regular sampling to maintain the population size roughly constant. A population of 2,000 (mostly young) cells were randomly sampled from the resulting population of cells, and simulated for an additional 40 generations. During this second simulation, all daughter cells produced from division are discarded, and snapshots are taken every 10 minutes, consistent with experimental procedures (Methods).

From those snapshots, single-cell level YFP concentration trajectories are obtained, and the trajectory of each individual cell is used to calculate the average expression level throughout the trajectory, along with two metrics of noise in the YFP expression level, the coefficient of variation (CV, σ/μ) and the Fano factor (σ^2/μ). For each of the above analysis, only the portion of the trajectory after the cell first reached ON state (defined as ≥ 5000 reporter molecules) is considered. The average of all individual cells (N > 1000) is calculated and used to generate Fig. 4 and Supplementary Fig. 3.

These results indicate that increasing r_{ON} or decreasing r_{OFF} increases expression level and vice versa, as expected. Further, having lower r_{ON} and r_{OFF} values generally increases the Fano factor, which is what one would expect, since slower bursts of activation results in larger bursts – reflected in the value of the Fano factor. On the other hand, r_{ON} has a much larger effect than r_{OFF} on the value of CV. We therefore tested the effects of varying r_{ON} alone, keeping r_{OFF}'s value fixed at the fitted value for the native GAL1 promoter (Fig. 4A-C, vertical dashed line). As can be seen in Supplementary Fig. 3, the average expression level steadily increases, and the CV steadily decreases, as r_{ON} increases. On the other hand, the Fano factor is non-monotonic, which we think is due to two competing factors: increasing r_{ON} values reduces the burst character (by making promoters spend more time in the ON state) of transcription, but at the same time increases the overall expression level.

Experimental measurements showed that the average expression level didn't increase during aging, however, so we further tested how simultaneously varying r_{ON} and r_{OFF} (at the same ratio so as to keep the average expression level constant) would affect the results. The results are shown in Fig. 4D-F.

To examine the behavior of the modified GAL1 promoter when r_{ON} and r_{OFF} are changed, we performed the same analyses starting from the parameter values obtained for the WP274 strain (Supplementary Fig. 5-7). As expected, compared to the simulations using the wild-type GAL1 promoter (Fig. 4), we see a lower level of noise generally, but the extent of noise reduction that can be achieved by varying the value of r_{ON} and r_{OFF} is significantly diminished, consistent with the experimental observations (Fig. 7).

The rates of transcription and translation, and the stability of their product, may change during aging. For instance, aging has been reported to translation and protein degradation rates to decrease in other organisms[5-7]. We computationally examined whether changes in those rates, without more, might account for the noise changes we observed. As the reporter protein in our experimental system, YFP, is extremely stable, and thus the effect from any active degradation would be miniscule compared to dilution effects from cell growth and division, we focused our efforts on the effects of changes in the transcription rate, the mRNA degradation rate, and the translation rate.

Keeping all promoter-dynamics parameters constant, we varied the mRNA synthesis and degradation rate by up to 10-fold in both directions from their original values in Supplementary Table 3 to explore the impact of their changes on noise (thus, range for the translation rate is between 0.15383 min^{-1} and 15.383 min^{-1}, while the range for the degradation rate is between 0.004077 min^{-1} and 0.4077 min^{-1}). In keeping with the experimental observation that the average expression level didn't change during aging, we adjusted the translation rate in each case so as to keep the expression level roughly constant (Supplementary Fig. 8, bottom panel). The resulting adjusted translation rate ranged from 0.08309 min^{-1} to 350.75 min^{-1}, compared to the original value of 4 min^{-1} (Supplementary Table 3). While we did observe slight noise decreases in some cases (Supplementary Fig. 8, top panels), they were not enough to account for the level of noise decrease observed experimentally.

Strain	Genotype
yTY10a (WT)	*MATα, his3Δ, leu2Δ, ura3Δ, met15Δ, ho*::*HIS5*-P$_{GAL1}$-YFP
WP190	*MATα, his3Δ, leu2Δ, ura3Δ, met15Δ, ho*::*HIS5*-P$_{GAL1}$-YFP, *gal80Δ*::*KanMX*
GESC19	*MATα, his3Δ, leu2Δ, ura3Δ, met15Δ, ho*::*HIS5*-P$_{GAL1}$-YFP, *rpd3Δ*::*KanMX*
GESC11	*MATα, his3Δ, leu2Δ, ura3Δ, met15Δ, ho*::*HIS5*-P$_{GAL1*}$-YFP
WP274	*MATα, his3Δ, leu2Δ, ura3Δ, met15Δ, ho*::*HIS5*-P$_{GAL1*}$-YFP, *gal80Δ*::*CaURA3*
WP268	*MATα, his3Δ, leu2Δ, ura3Δ, met15Δ, ho*::*HIS5*-P$_{HHF2}$-YFP

Supplementary Table 1. *Saccharomyces cerevisiae* strains used in this study. All *S. cerevisiae* strains used have the haploid BY genetic background. P$_{GAL1*}$ indicates the synthetic version of the *GAL1* promoter (carrying the nucleosome disfavoring sequences). Strains WP190 and GESC19 were referred to as strains '*gal80Δ*' and '*rpd3Δ*', respectively, in the manuscript.

Parameter	Meaning	Value	Unit
mean and SD of r_1	Growth rate, G1	0.216 ± 0.054	fL min^{-1}
mean and SD of r_2	Growth rate, S/G2/M, total	0.45477 ± 0.097236	fL min^{-1}
mean and SD of r_{2m}	Growth rate, S/G2/M, mother compartment	0.0063 ± 0.0216	fL min^{-1}
mean and SD of *T1'*	Minimum time before *start* for mothers	13 ± 6.968	min
mean and SD of *T2*	Time between *start* and S phase entry	16 ± 4.992	min
mean and SD of *T3*	Duration of S/G2/M	55.75 ± 6.467	min
mean and SD of V_i	Initial volume	25 ± 5	fL
k	Constants relating volume at *start* with r_1	116	min
b		11.0	fL
c	Level of inheritance for daughters	0.25	

Supplementary Table 2. Parameters for the volume module.

Parameter	Meaning	Value	Unit	References & Notes
$r'_{m,\ GAL3}$	Apparent maximum transcription rate, GAL3	0.7223	min^{-1}	[Note A] [Note J]
$r'_{m,\ GAL80}$	Apparent maximum transcription rate, GAL80	0.1394	min^{-1}	[Note B] [Note J]
$r'_{m,\ GAL1}$, $r'_{m,\ PGAL1\text{-}YFP}$	Apparent maximum transcription rate, GAL1	1.5383	min^{-1}	[Note C] [Note J]
$r_{p,\ GAL3}$	Translation rate, Gal3p	20	min^{-1}	[Note A]
$r_{p,\ GAL80}$	Translation rate, Gal80p	4	min^{-1}	[Note B]
$r_{p,\ GAL1}$	Translation rate, Gal1p	20	min^{-1}	[Note D]
$r_{p,\ PGAL1\text{-}YFP}$	Translation rate, YFP	4	min^{-1}	[Note E]
$d_{m,\ GAL3}$	Degradation rate, Gal3 mRNA	0.0330	min^{-1}	[8]
$d_{m,\ GAL80}$	Degradation rate, Gal80 mRNA	0.02567	min^{-1}	[8]
$d_{m,\ GAL1}$	Degradation rate, Gal1 mRNA	0.04077	min^{-1}	[8]
$d_{m,\ PGAL1\text{-}YFP}$	Degradation rate, YFP mRNA	0.04077	min^{-1}	[Note F]
$d_{p,\ GAL3}$	Degradation rate, Gal3p	0.03851	min^{-1}	[9]
$d_{p,\ GAL80}$	Degradation rate, Gal80p	0.00024	min^{-1}	[9]
$d_{p,\ GAL1}$	Degradation rate, Gal1p	0.00048135	min^{-1}	[Note G][10]
$d_{p,\ PGAL1\text{-}YFP}$	Degradation rate, YFP	0.00048135	min^{-1}	[Note G]
b'_{GAL1}, $b'_{PGAL1\text{-}YFP}$	Apparent basal transcription level, GAL1	0.0018		[Note J][11]
b'_{GAL3}	Apparent basal transcription level, GAL3	0.07294		[Note J][11]
b'_{GAL80}	Apparent basal transcription level, GAL80	0.21739		[Note J][11]
S_{80}	Scale of action, Gal80p	30000		[Note H]
α	Nonlinearity of Gal80p-Gal3p/Gal1p interaction	1		[12]
V_{ref}	Average volume of entire population	50	fL	[Note I]

Supplementary Table 3. Fixed parameters for the gene network module.

[Note A] Calculated using a basal level of 721 Gal3p/cell[13], basal transcription level[11] and protein half-life[9] from literature, and a translation rate of 20/mRNA/min.

[Note B] Calculated using a basal level of 784 Gal80p/cell[13], basal transcription level[11] and protein half-life[9] from literature, and a translation rate of 4/mRNA/min.

[Note C] Calculated from estimated mRNA abundance based on literature[11] and mRNA half-life measurement in galactose[8].

[Note D] Assigned based on estimated average translation rate[9,13,14] in yeast. Inaccuracies in this rate constant are accounted while fitting the scale parameter S_1.

[Note E] Arbitrarily assigned. Any inaccuracy is accounted for during the fluorescence fitting process.

[Note F] The degradation rate is assumed to be similar to that of GAL1 mRNA. Any inaccuracy is accounted for during the fluorescence fitting process.

[Note G] This protein is highly stable and assigned a half-life of 24 hours.

[Note H] Arbitrarily assigned.

[Note I] Assigned based on the results from running the volume model.

[Note J] Applicable to wild-type promoter only. It is assumed that the actual transcription rate from the ON and OFF state promoter do not change; however, changes in the maximum fraction of time the promoter is in the ON state will lead to changes in the apparent maximum transcription rate (defined as the transcription rate measured at full induction).

Parameter	Meaning	Fitted value	Unit	Initial value	Lower bound	Upper bound
$r_{ON,\ GAL1}$ $r_{ON,\ PGAL1\text{-}YFP}$	Maximum OFF-to-ON transition rate	0.0168	min^{-1}	0.1	0.01	10
f_{GAL1} $f_{PGAL1\text{-}YFP}$	Fraction of time the promoter is in the ON state at maximum induction	0.9483		0.5	0.01	0.99
u	Fluorescence (a.u.) per YFP protein	0.0153		1	0.0001	10000

Supplementary Table 4. Fitted parameters for the WP274 strain.

Parameter	Meaning	Value	Unit	Notes
$r'_{m,\ PGAL1\text{-}YFP}$	Apparent maximum transcription rate, P_{GAL1}-YFP	3.6727	min^{-1}	The apparent transcription rate is calculated by multiplying the apparent transcription rate of the wild type GAL1 promoter (Supplementary Table 3) by the ratio of the expression level in the WP274 and *gal80Δ* strains. The apparent basal transcription level is then calculated on the assumption that the actual transcription rates from ON and OFF state do not change. Hence, increasing the *apparent* maximum transcription rate will decrease the *apparent* basal expression level (whose value is calculated by dividing the transcription rate of the OFF state with the apparent maximum transcription rate).
$b'_{PGAL1\text{-}YFP}$	Apparent basal transcription level, P_{GAL1}-YFP	0.00075		

Supplementary Table 5. Additional fixed parameters for the WP274 strain.

Parameter	Meaning	Fitted value	Unit	Initial value	Lower bound	Upper bound
$r_{ON,\ GAL1}$ $r_{ON,\ PGAL1\text{-}YFP}$	Maximum OFF-to-ON transition rate	0.0229	min^{-1}	0.14	0.01	10

Supplementary Table 6. Fitted parameter for the *gal80Δ* strain.

Parameter	Meaning	Value	Unit	Notes
f_{GAL1} $f_{PGAL1\text{-}YFP}$	Fraction of time the promoter is in the ON state at maximum induction	0.3972		This value is calculated from the fitted *f* value of the WP274 strain and the expression level differences between the two strain (as determined experimentally).

Supplementary Table 7. Additional fixed parameter for the *gal80Δ* strain.

Parameter	Meaning	Fitted value	Unit	Initial value	Lower bound	Upper bound
$r_{ON,\ GAL3}$	Maximum OFF-to-ON transition rate	0.01	min^{-1}	0.01	0.01	10
$r_{ON,\ GAL80}$		0.3972	min^{-1}	0.2	0.01	10
f_{GAL3}	Fraction of time the promoter is in the ON state at maximum induction	0.8477		0.9	0.01	0.99
f_{GAL80}		0.9517		0.9	0.01	0.99
S_3	Scale of action, Gal3p	22032.2		20000	4000	40000
S_1	Scale of action, Gal1p	9934.8		8000	20	10000
β	Nonlinearity of Gal80p-Gal4p interaction	4.2413		6	1	6

Supplementary Table 8. Fitted parameters from the WT strain.

Parameter	Meaning	Fitted value	Unit	Initial value	Lower bound	Upper bound
$r_{ON,\ GAL3}$	Maximum OFF-to-ON transition rate	0.1608	min^{-1}	0.01	0.01	10
$r_{ON,\ GAL80}$		0.0426	min^{-1}	0.3972	0.01	10
$r_{ON,\ GAL1}$<br>$r_{ON,\ PGAL1\text{-}YFP}$		0.0533	min^{-1}	0.0229	0.01	10
f_{GAL3}	Fraction of time the promoter is in the ON state at maximum induction	0.9485		0.8477	0.01	0.99
f_{GAL80}		0.9882		0.9517	0.01	0.99
f_{GAL1}<br>$f_{PGAL1\text{-}YFP}$		0.3935		0.3972	0.01	0.99

Supplementary Table 9. Fitted parameters for the *rpd3Δ* strain.

Parameter	Meaning	Value	Unit	Notes
$r'_{m,\ GAL3}$	Apparent maximum transcription rate	0.8019	min^{-1}	These values are calculated based on the assumption that the actual transcription rates from ON and OFF state do not change. Hence, increasing *f* (spending more time in ON state) will increase the *apparent* maximum transcription rate and decrease the *apparent* basal expression level, and vice versa.
$r'_{m,\ GAL80}$		0.1436	min^{-1}	
$r'_{m,\ GAL1,}$<br>$r'_{m,\ PGAL1\text{-}YFP}$		1.5240	min^{-1}	
b'_{GAL1}, $b'_{PGAL1\text{-}YFP}$	Apparent basal transcription level	0.0657		
b'_{GAL3}		0.2111		
b'_{GAL80}		0.0018		

Supplementary Table 10. Parameters calculated from fitted parameters for the *rpd3Δ* strain.

SUPPLEMENTARY REFERENCES

1 Peng, W., Song, R. & Acar, M. Noise reduction facilitated by dosage compensation in gene networks. *Nat Commun* **7**, 12959, doi:10.1038/ncomms12959 (2016).

2 Ferrezuelo, F. *et al.* The critical size is set at a single-cell level by growth rate to attain homeostasis and adaptation. *Nat Commun* **3**, 1012, doi:10.1038/ncomms2015 (2012).

3 Nelder, J. A. & Mead, R. A Simplex Method for Function Minimization. *The Computer Journal* **7**, 308-313, doi:10.1093/comjnl/7.4.308 (1965).

4 Johnson, S. G. *The NLopt nonlinear-optimization package*, <http://ab-initio.mit.edu/nlopt> (2014).

5 Liang, V. *et al.* Altered proteostasis in aging and heat shock response in C. elegans revealed by analysis of the global and de novo synthesized proteome. *Cellular and Molecular Life Sciences* **71**, 3339-3361, doi:10.1007/s00018-014-1558-7 (2014).

6 Kirstein-Miles, J., Scior, A., Deuerling, E. & Morimoto, R. I. The nascent polypeptide-associated complex is a key regulator of proteostasis. *The EMBO Journal* **32**, 1451-1468, doi:10.1038/emboj.2013.87 (2013).

7 Sharma, H. K., Prasanna, H. R., Lane, R. S. & Rothstein, M. The effect of age on enolase turnover in the free-living nematode, Turbatrix aceti. *Archives of Biochemistry and Biophysics* **194**, 275-282, doi:10.1016/0003-9861(79)90619-2 (1979).

8 Munchel, S. E., Shultzaberger, R. K., Takizawa, N. & Weis, K. Dynamic profiling of mRNA turnover reveals gene-specific and system-wide regulation of mRNA decay. *Molecular Biology of the Cell* **22**, 2787-2795, doi:10.1091/mbc.E11-01-0028 (2011).

9 Belle, A., Tanay, A., Bitincka, L., Shamir, R. & O'Shea, E. K. Quantification of protein half-lives in the budding yeast proteome. *Proceedings of the National Academy of Sciences of the United States of America* **103**, 13004-13009, doi:10.1073/pnas.0605420103 (2006).
10 Zacharioudakis, I., Gligoris, T. & Tzamarias, D. A Yeast Catabolic Enzyme Controls Transcriptional Memory. *Current Biology* **17**, 2041-2046, doi:10.1016/j.cub.2007.10.044 (2007).
11 Hsu, C. *et al.* Stochastic signalling rewires the interaction map of a multiple feedback network during yeast evolution. *Nat Commun* **3**, 682, doi:10.1038/ncomms1687 (2012).
12 Timson, D. J., Ross, H. C. & Reece, R. J. Gal3p and Gal1p interact with the transcriptional repressor Gal80p to form a complex of 1:1 stoichiometry. *Biochemical Journal* **363**, 515-520, doi:10.1042/0264-6021:3630515 (2002).
13 Ghaemmaghami, S. *et al.* Global analysis of protein expression in yeast. *Nature* **425**, 737-741, doi:10.1038/nature02046 (2003).
14 To, T.-L. & Maheshri, N. Noise Can Induce Bimodality in Positive Transcriptional Feedback Loops Without Bistability. *Science* **327**, 1142-1145, doi:10.1126/science.1178962 (2010).
